# Identification of Promising Drug Candidates against Prostate Cancer through Computationally-Driven Drug Repurposing

**DOI:** 10.3390/ijms24043135

**Published:** 2023-02-05

**Authors:** Leonardo Bernal, Luca Pinzi, Giulio Rastelli

**Affiliations:** 1 Department of Life Sciences, University of Modena and Reggio Emilia, Via Giuseppe Campi 103, 41125 Modena, Italy; 2 Clinical and Experimental Medicine PhD Program, University of Modena and Reggio Emilia, 41125 Modena, Italy

**Keywords:** prostate cancer, drug repurposing, chemoinformatics

## Abstract

Prostate cancer (PC) is one of the most common types of cancer in males. Although early stages of PC are generally associated with favorable outcomes, advanced phases of the disease present a significantly poorer prognosis. Moreover, currently available therapeutic options for the treatment of PC are still limited, being mainly focused on androgen deprivation therapies and being characterized by low efficacy in patients. As a consequence, there is a pressing need to identify alternative and more effective therapeutics. In this study, we performed large-scale 2D and 3D similarity analyses between compounds reported in the DrugBank database and ChEMBL molecules with reported anti-proliferative activity on various PC cell lines. The analyses included also the identification of biological targets of ligands with potent activity on PC cells, as well as investigations on the activity annotations and clinical data associated with the more relevant compounds emerging from the ligand-based similarity results. The results led to the prioritization of a set of drugs and/or clinically tested candidates potentially useful in drug repurposing against PC.

## 1. Introduction

Prostate cancer (PC) is one of the most common types of tumors in men, with the number of diagnosed cases being significantly increased in recent years [1]. PC is particularly diffused in countries where advanced screening protocols (e.g., prostate-specific antigen (PSA) detection, MRI, and prostate biopsy) are available [2], accounting for more than 350,000 deaths per year [3]. According to recent studies, the prognosis and survival rates of PC patients heavily depend on tumor grade and the stage encountered at the primary diagnosis [4]. In particular, localized PC (~80% of the cases) presents a 5-year survival rate more than 90% [5], while more aggressive metastatic castration-sensitive (mCSPC) and castration-resistant PC (mCRPC) present a significantly lower survival rate (i.e., around 60–80% for locoregional metastases and around 30–40% for distant metastases) [6]. 

Standard early-stage PC therapy includes interventions, such as radiotherapy (RT) or radical prostatectomy, in combination with androgen deprivation therapy (ADT) or pelvic lymph node dissection (PLND) for patients with high-risk localized PC [7,8]. More aggressive late-stage prostate cancers are generally treated with ADT, albeit very often obtaining low therapeutic efficacy. Indeed, ADT is generally administered to PC patients on a lifelong basis in combination with Docetaxel (or its second-generation derivative Cabazitaxel) [9], drugs as androgen pathway inhibitors [10], or poly(ADP-ribose) polymerase inhibitors (PARPi) [11], considering the role of the androgen receptor (AR) for the tumor proliferation in mCRPC [12]. Additional androgen pathway inhibitors, such as abiraterone (ABI), enzalutamide (ENZ), or apalutamide (APA), have also recently been approved [11]. Moreover, new PARPi such as Olaparib and Rucaparib recently entered clinical use for mCRPC forms with germline alterations (i.e., BRCA2 mutation) [13]. Despite these recent developments, there still is a pressing need for the development of novel therapeutic agents, especially for the treatment of advanced stages of PC [14].

Considering that de novo drug discovery usually: (i) presents low success rates (~2%) [15], which are typically due to efficacy or safety issues [16,17]; (ii) requires a significant amount of resources and investments [18]; (iii) requires several years to deliver a new drug to the market, other strategies have been proposed to more efficiently identify effective therapeutics. Among them, drug repurposing (also known as drug repositioning) emerged as a valuable alternative or complementary approach [19], being able to potentially circumvent issues typically associated with de novo drug discovery [20,21,22]. The integration of different in silico approaches and workflows has been demonstrated to provide significant advantages for the identification of new drug repurposing possibilities [23]. Such data-driven approaches have been fueled by the constant increase in structural, biological, and chemical information reported into public repositories, such as DrugBank [24], ChEMBL [25], PubChem [26], UniProt [27], Therapeutic Target Database (TTD) [28], and clinicaltrials.gov [29,30,31,32,33]. Although many attempts to integrate such data have been proposed with success in this context [34,35,36], such information remains often scattered among various sources, complicating drug repurposing approaches.

On these premises, in this work, we aimed to identify potential drug repurposing candidates via an integrated, ad hoc in silico approach. In particular, we devised a repurposing workflow based on extensive ligand-based similarity estimations between DrugBank [24] compounds and a set of molecules with potent activity on PC cells retrieved from ChEMBL [25] ligands, these databases being widely employed in in silico drug discovery repurposing research [34,37,38].

We selected compounds from DrugBank for the analyses as it contains freely available data related to FDA-approved drugs, and pre-clinical and clinical candidates, including information on their properties, mechanisms of action, interactions with different molecules, pathways, and information on clinical trials [24]. Compounds with potent activity on PC cells were collected from ChEMBL, which is one among the most large, open-access resources of molecules with manually curated bioactivity data on targets and cell lines currently available, reporting also information about properties, pharmacokinetics, and safety profiles of ligands. 

The adopted approach allowed us to identify a set of DrugBank compounds significantly similar to molecules with known potent PC anti-proliferative activity. Moreover, the analyses highlighted biological targets of ligands with reported potent activity on PC cells, which could be potentially relevant for their antiproliferative activity. Overall, the analyses on activity annotations and clinical data allowed us to confirm five top-scoring drugs that are already in clinical trials against PC, and to identify ten drugs, already assessed at various preclinical and clinical stages for different pathologies, which could be considered as valuable candidates for fast repurposing against PC. 

## 2. Results and Discussion

**Figure 1:**
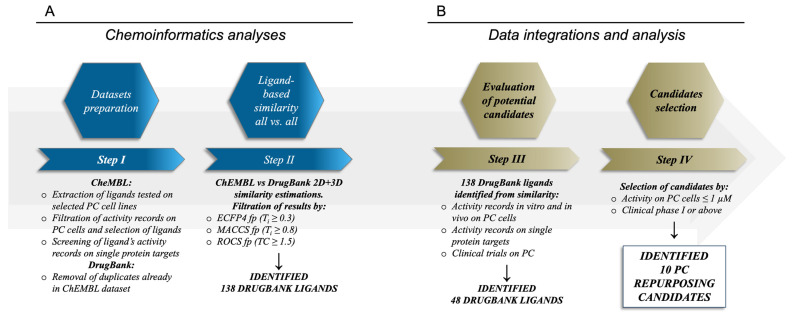
Workflow adopted for the identification of potential drug repurposing candidates for PC. Panel (**A**) describes the ligand-based procedure, while panel (**B**) contains the data integration and analysis procedure for the selection of the repurposing candidates.

Therapeutic options for advanced-stage prostate cancer are still limited and very often not effective [12]. Considering the extended timelines usually required for the development and approval of novel drugs, drug repurposing approaches, especially when driven by in silico methods, can represent faster yet valuable alternatives or complement methods for the identification of novel therapeutic treatments against PC, similarly to what is already observed for other diseases [21,22,30,31,32,33]. On these premises, we devised and applied a computationally driven drug repurposing workflow (Figure 1) in the search for novel drug candidates potentially suitable for PC therapy. 

Moreover, a series of analyses were also conducted on the annotations of ChEMBL compounds with reported PC activity, in the search for molecular targets that are more likely to be involved in the observed cell-based antiproliferative effects (see below).

### 2.1. Analyses of the Compounds in the ChEMBL Dataset and of Their Target Activity Data

A set of ChEMBL ligands tested also on PC cells was first curated as detailed in the Methods section. The majority of these ligands derive from experiments on the PC-3, DU-145, and LNCaP cells (Table S1). Of note, most of the reported activity annotations of these compounds were below 1 µM (i.e., “highly active” records) in at least one PC cell line, while the remaining annotations followed a decreasing trend toward higher values (Figure S1 and Table S2). Moreover, the compounds showed a good degree of variability in terms of LogP, PSA, HBD, HBA, MW, volume, and ovality molecular descriptors, with distributions similar to those evaluated for the DrugBank ligands under investigation (Figure S2). In particular, most of the compounds in the ChEMBL dataset presented acceptable solubility and good membrane permeability, with LogP in the range of 2–4, and PSA values in the range of 50–100 Å^2^ [39]. Moreover, most of the investigated molecules presented 1 or 2 HBD and 3 to 6 HBA, which is in line with *“Lipinski’s rule of five”* [40]. The distribution of molecular weight, volume, and ovality, which are three indicators of small and spherical-shaped molecules, resulted in the 200–450 Da, 200–400 Å^3^, and 0.65–0.75 ranges, respectively. Moreover, comparison of these physicochemical properties highlighted a significant overlap between the properties of compounds in the two curated datasets (i.e., ChEMBL and DrugBank datasets, Figure S2), which is interesting in the view of repositioning molecules employing in silico similarity estimations.

**Figure 2:**
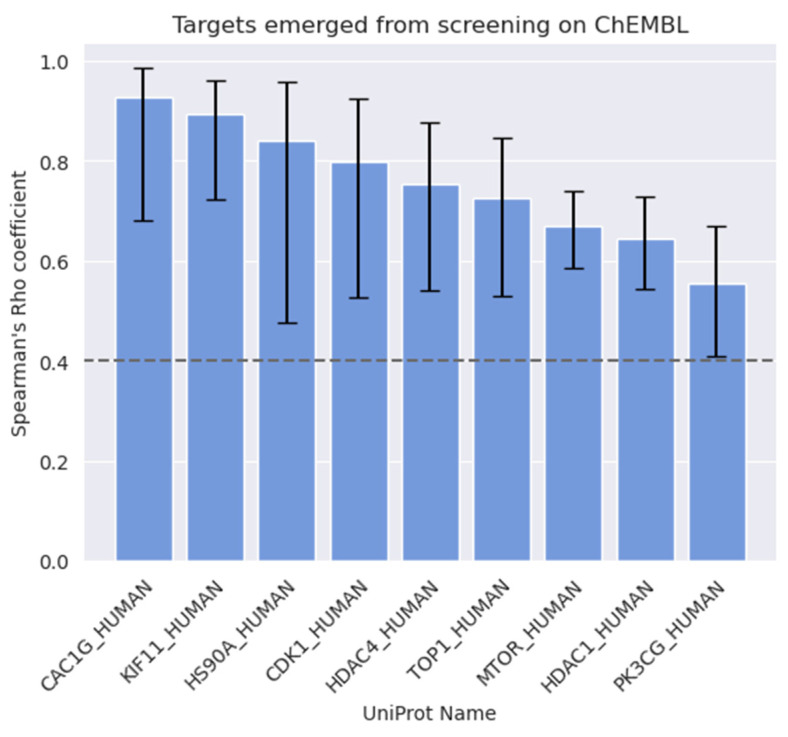
Targets whose activity data correlate with antiproliferative activity on PC cells (*ρ_s_* ≥ 0.4, *p*-value < 0.001).

Besides the investigations on key molecular descriptors, analyses of the target activity annotations of the ChEMBL datasets were also performed. These analyses aimed at identifying correlations between activity on biological targets and antiproliferative effects on different PC cell lines. To this aim, target annotations were first filtered and processed as described in the Methods section. Targets with fewer than ten activity records were not considered in the analysis for statistical robustness. Moreover, the analyses were restricted to compounds with reported antiproliferative activity on PC-3, DU-145, and LNCaP cell lines, which are the most widely studied in prostate cancer research (Tables S1 and S2; Figure S3). Indeed, a broad spectrum of PC cell lines has been studied over the past decades to characterize cellular mechanisms and aberrations at a molecular level responsible for development and progression of different subtypes of prostate cancer [41]. However, major interest has been placed on PC-3, DU-145, and LNCaP cell lines due to their ability to represent prostate cancer with different levels of aggressiveness, metastatic potential and hormonal dependency. In particular, PC-3 are cells collected from a lumbar vertebral metastasis of advanced-staged prostate cancer, showing high metastatic potential and aggressiveness, and low androgen dependency [42]. DU-145 are androgen-refractory cells collected from central nervous system (CNS) metastases of prostate cancer, with moderated metastatic potential [43]. LNCaP are androgen-dependent cells with low metastatic potential, which were collected from lymph node metastases of PC [44]. Compounds tested on PC-3 and DU-145 cells revealed target activities with a good degree of correlation with antiproliferative cellular activity (*Spearman’s Rho ρ_s_* ≥ 0.4, *p-*value < 0.001), whereas targets with a statistically significant correlation to cellular activity against LNCaP cells were not found, most likely because of the limited amount of data available for this cell line compared to PC-3 and DU-145 cells. The performed analyses allowed us to highlight a total of 9 biological targets with significant correlation (see Table 1 and Figure 2) with PC antiproliferative activity. The complete list of the targets investigated in this study is reported in Tables S3 and S4 and Figure S4.

Interestingly, all the targets reported in Figure 2 have been previously studied for their involvement in PC development and progression. For example, mTOR and PI3Kγ emerged as two of the most relevant targets for PC-3 treatment, regulating the PI3K/AKT/mTOR signaling pathway, and both have been found to be significantly overexpressed in several types of cancers, including PC [45,46]. Of note, the PI3K/Akt/mTOR signaling cascade is one of the most studied for the treatment of PC [47], for which novel therapeutic agents are being developed [48]. 

Heat Shock Protein 90 (Hsp90) is a chaperone protein with a recognized role in the regulation of folding and degradation of many oncogenic signaling proteins. Several preclinical and clinical studies assessing Hsp90 inhibitors have been reported so far, although none of them have received approval for therapeutic use to date [49,50,51]. Interestingly, Hsp90 has recently come to light again as a promising anti-cancer target against several types of tumors including PC, especially in multi-target therapies [52,53]. In addition, we also identified two members of the Histone deacetylase family (i.e., HDAC1 and HDAC4), which are known to be key regulators of chromatin structure and post-translation modifiers on several proteins in different tissues [54]. In particular, HDAC1 is a class I HDAC member that plays a crucial role in cellular epigenetic landscaping and transcriptional repression [55,56]. HDAC1 pathological expression is recognized in different types of cancers including PC [57], where its overexpression increases proliferation and differentiation [55,58,59,60,61], making it an interesting therapeutic target for several diseases. HDAC4 is a class IIA HDAC that exhibits tissue-specific patterns of expression. Alterations of HDAC4 activity, such as its overexpression and its repression effects on the tumor-suppressor gene p21, are well documented in different types of tumors [62,63,64,65,66], with its role in PC development and survival being reported in the literature [67,68]. 

Kinesin family member 11 (KIF11) is a molecular motor protein essential in mitosis and its inactivation causes cell cycle arrest and apoptotic cell death [69]. This protein also has a central role in several cancers [70,71,72,73], and its overexpression is a biomarker that could be associated with poor outcomes [74,75]. Of note, several studies suggest that KIF11 expression levels might be related to PC aggressiveness and bone metastasis occurrence, with this protein being potentially involved in the metastatic process [76,77]. Various KIF11 inhibitors are currently under development (i.e., Ispinesib, *S*-(methoxytrityl)-L-cysteine, Monastrol) for the treatment of tumors including prostate cancer [78,79,80,81]. Although the modulation of KIF11 did not result in the expected therapeutic efficacy in some cases [82], it remains an interesting target for PC with a proven prognostic and a potential therapeutic role [83,84]. 

T-type Ca^2+^ channels are transporters for Ca^2+^, which is a fundamental requirement for tumor progression and proliferation [85,86,87,88]. One of the three isoforms of T-type Ca^2+^ channels is Cav3.1 (also known as α1G), which is expressed in different cancers, such as breast cancer [89], small-cell lung cancer [90], and retinoblastoma [91]. Moreover, Cav3.1 downregulation by complementary shRNA has been recently observed to lead to a decrease in cell growth and migration, through the targeting of the AKT signaling in PC cells. These results open to the possibility that Cav3.1 may be a novel potential target for PC treatment [92,93]. 

Cyclin-Dependent Kinase 1 (CDK1, also known as p34, or CDC2) is a promoter of cell cycle progression, which is deeply involved in cell growth progression [94]. CDK1 activity is also linked to apoptosis induction in different pathways [95]. Moreover, it has been reported to play a central role in different types of cancers [96,97,98,99,100,101,102,103]. CDK1 has also a role in the regulation of AR phosphorylation and expression, suggesting a potential enhancement in the response to androgen deprivation therapy through CDK1 inhibition [104,105]. 

DNA topoisomerase I (i.e., TOP1) is a ubiquitous enzyme involved in the relaxation of DNA supercoiling, which is often targeted, along with topoisomerase II, by a wide variety of antimicrobial and anticancer agents [106,107]. Moreover, the inhibition of TOP1 with consequent DNA damage and apoptosis induction has been reported in PC cells [108,109,110,111]. Besides the ones described above, additional targets showing correlation coefficients slightly below the selected threshold were also identified (see Table S4 and Figure S4) such as PI3Kα/β [48,112], Androgen Receptor [113], Glycogen Synthase Kinase-3 β [114], STAT3 [115], and Serine/Threonine-Protein Kinase Aurora-A [116], which, however, present a recognized role in PC development and progression. These targets showed lower correlations to PC antiproliferative activity most likely because they lack exhaustive target activity annotations in ChEMBL.

### 2.2. Similarity Estimations and Selection of the Candidates for Drug Repurposing 

Extensive 2D MACCS and ECFP4 fingerprints-based, followed by 3D-shape-based, similarity estimations with ROCS (Rapid Overlay of Chemical Structures, OpenEye) [117] were performed between the DrugBank and ChEMBL datasets, as detailed in the Methods section. These analyses were performed to identify DrugBank ligands already assessed at pre-clinical and clinical stages similar to ChEMBL compounds with known activity against prostate cancer cell lines. The performed similarity analyses highlighted an overall low degree of similarity between the compounds in the two datasets, which is a consequence of the high diversity between the chemical scaffolds of the ligands considered (Figure S5). However, these analyses allowed us to identify 138 DrugBank ligands with a high degree of similarity with molecules having antiproliferative activity on PC cells below 1 µM, according to both 2D and 3D similarity indexes (see Table S5). The 138 identified DrugBank ligands (Figure 1, panel A) included molecules investigated for their efficacy and toxicity at preclinical levels, and more importantly drug candidates already tested in clinical settings, with these latter being the most interesting in the view of fast repurposing against PC. In addition, the identified DrugBank compounds have also reported activity data on biological targets and on cell lines that were in a number of cases already tested against prostate cancer cells. On these premises, the identification of the best candidates for drug repurposing on PC required a further step of integration and analysis of their literature data (Figure 1, panel B). In particular, our attention focused on: (i) in vitro activity data records on PC cells; (ii) in vivo activity data records on PC; (iii) clinical trials on PC or solid tumors with PC patients; (iv) bioactivity records on targets with a proven relation to PC. Of note, the performed analyses allowed us to provide a validation of the adopted workflow (see below), and more importantly to identify 48 DrugBank compounds that meet at least one or more of the above-mentioned criteria, which could be, therefore, considered of high interest for their potential repurposing against PC. The list of the identified 48 DrugBank ligands with their similarity statistics is reported in Table S6. 

Of the 48 DrugBank ligands, 10 molecules with reported activity on PC cells lower than 1 µM, and whose safety profile had already been assessed at least in a phase I study, were selected as the most suitable candidates for fast repurposing against PC (Table 2). Interestingly, some of the identified ligands have been previously included in trials including PC patients, although this type of cancer was not the main focus of these studies. Moreover, some of these compounds have also been reported to be active on targets relevant to PC (e.g., Vistusertib, Bimiralisib, Onatasertib, VS-5584, and BIIB021 in Table 2) and could exert potential beneficial polypharmacological effects. Of note, 5 of the 48 identified DrugBank molecules have already been assessed in clinical trials for PC as a single agent or as a combination of drugs (underlined in Table 2). These molecules have been assessed in phase I/II clinical trials, together with other therapeutic agents, such as Docetaxel or Androgen Receptor inhibitors (e.g., LAE001) to assess their overall efficacy and potentially unwanted side-effects when used in combination. The remaining 33 ligands were excluded at the final stage of candidates’ selection, due to the poor activity on PC cells, lack of novelty of the molecules, or toxicity in humans. Information related to these latter ligands is reported in Table S7. A detailed discussion of the drug candidates and the molecules already assessed in PC trials is reported below (i.e., “*Molecules already under clinical evaluation for PC*” and “*New potential Candidates for drug repurposing*”). 

### 2.3. Molecules Already under Clinical Evaluation for PC

An analysis of literature data reported for the selected candidates highlighted five molecules that have already been included in clinical trials for prostate cancer therapy, either as single agents or in combination with other drugs (Table 2). To some extent, this result provides an internal validation of the applied methodology and raised interesting considerations regarding their enrollment in PC clinical trials. In particular, **Azacitidine (DB00928)** is a cytosine analog and hypomethylating agent, which is used as an antineoplastic agent in the therapy of myelodysplastic syndromes [132]. Azacitidine efficacy as a single agent was assessed in a phase II clinical trial on chemo-naive CRPC patients, resulting in favorable disease-modifying activities [119]. Azacitidine has also entered a phase I/II clinical trial (clinicaltrials.gov ID: NCT00503984), in combination with docetaxel and prednisone in mCRPC patients previously treated with docetaxel-based therapy, albeit conclusive results have not been delivered yet.

**Trimetretexate (TMTX, DB01157)** is a lipophilic inhibitor of DHFR that has been approved for the management of *Pneumocystis jiroveci* pneumonia in patients with AIDS [140]. TMTX efficacy against advanced hormone-refractory prostate cancer was assessed in a phase II trial in the early 1990s, although its clinical usefulness for PC treatment resulted as limited [120]. 

**Afuresertib (GSK2110183, DB11648)** is a pan-AKT kinase inhibitor with low nanomolar potency against AKT2 (IC_50_ = 10 nM) [141], AKT3 (IC_50_ = 16.9 nM) [141], and AKT1 (IC_50_ = 1 nM) [141], with the latter target being often hyperactivated in mCRPC [142]. Afuresertib has been demonstrated to inhibit the proliferation of various cancer cells, with a particular effect on hematological diseases [121]. More importantly, Afuresertib has recently been included in phase I/II trials against mCRPC in combination with LAE001, which is an androgen synthesis inhibitor, and Prednisone (clinicaltrials.gov ID: NCT04060394), providing promising results [143].

**Vistusertib (AZD2014, DB11925)** is an orally bioavailable, low-nanomolar inhibitor of mTOR (IC_50_ = 2.8 nM) [144], which demonstrated promising antineoplastic activity [145,146,147]. Vistusertib has also shown low nanomolar activity against PI3Kα (K_d_ = 33 nM) [148], which is a target implicated in the PI3K/AKT/mTOR signaling pathway, recognized to play a role in PC development and progression [46]. Vistusertib has also been enrolled in clinical trials for prostate cancer patients prior to radical prostatectomy (clinicaltrials.gov ID: NCT02064608) and in patients with CRPC in combination with AZD8186 (clinicaltrials.gov ID: NCT01884285). The results of these studies suggest that Vistusertib is tolerated in humans and inhibits mTOR1/2 in tumor tissues [149]. Preliminary results showed efficacy when it is used in combination with AZD8186 on a CRPC patient, with the study of the safety and tolerability of this combination still in place [150].

**Exatecan (DB12185)** is a novel camptothecin derivative and a TOP1 inhibitor, which has shown antiproliferative effects against various cancer cell lines [151]. In a phase II trial, DX-8951F (Exatecan mesylate) effectiveness was assessed for the treatment of patients with metastatic prostate cancer that has not responded to hormone therapy (clinicaltrials.gov ID: NCT00004045); however, clinical development has been discontinued.

### 2.4. New Potential Candidates for Drug Repurposing

Besides compounds already enrolled in clinical trials against prostate cancer, we also identified 10 compounds with tested safety profiles, significant activity on PC cell lines, and more advanced experimentation. The molecules reported in the lower part of Table 2 represent valuable putative candidates for fast repurposing against PC. Interestingly, some of these molecules previously entered clinical trials for their therapeutic assessment against different types of cancers and they have been demonstrated to exert their therapeutic effects through the modulation of targets and pathways highly relevant also in PC. Moreover, some of the compounds have already been tested in trials focusing on solid tumors, also including few PC patients, and have associated bioactivity data on several cell lines of different tumors (Table S8). In addition, some of the identified compounds have been clinically tested or approved in completely different therapeutic areas and represent candidates in which more novelty comes into play.

One among the most promising ligands of this class is **Bimiralisib (PQR-309, DB14846)**, which has been reported to potently inhibit the *α*, β, and γ isoforms of kinase PI3K (IC_50_ = 33 nM, IC_50_ = 661 nM, and K_d_ = 25 nM, respectively), and mTOR (IC_50_ = 89 nM) [123]. Interestingly, both mTOR and PI3Kγ are two of the targets that demonstrated a significant correlation with the activity on PC cells, based on the analyses conducted on the activity records obtained from ChEMBL (see Table 1). The ability of this compound to inhibit different key players of the PI3K/AKT/mTOR signaling cascade is of particular relevance for PC treatment, with this signaling pathway being one of the most commonly disrupted in cancer, and being crucial for cell motility, growth, survival, metabolism, and the establishment of drug resistance [46,152]. Furthermore, inhibitors of the PI3K/mTOR/AKT pathway have been proven to be effective against several different PC cells [153,154], including DU-145. Bimiralisib is in phase II clinical trials for the treatment of head and neck cancer (clinicaltrials.gov ID: NCT03740100), glioblastoma (clinicaltrials.gov ID: NCT02850744), lymphoma (clinicaltrials.gov ID: NCT02249429), and breast cancer (clinicaltrials.gov ID: NCT02723877). Importantly, this compound has demonstrated potent antiproliferative effects on PC-3 mice xenografts, and it also has low nanomolar activity on mTOR and sub-micromolar activity on PI3Kγ (Table 2). Moreover, it can also cross the blood–brain barrier (BBB) [155], making it one of the most promising molecules for drug repurposing on PC, especially for treatment of PC patients with brain metastases. Based on the safety profile of this molecule, specific clinical trials on PC could reveal its potential as a therapeutic agent for this disease.

**Onatasertib (CC-223, DB12570)** is an orally available, low nanomolar inhibitor of mTOR (IC_50_ = 10 nM) [156], with putative antineoplastic activity [133,157,158]. The inhibition of mTOR by Onatasertib has been reported to induce tumor cell apoptosis and to decrease tumor cell proliferation [124,159,160,161]. Onatasertib is currently in phase II trials for the treatment of multiple myeloma, non-Hodgkin’s lymphoma (clinicaltrials.gov ID: NCT01177397), and hepatocellular carcinoma (clinicaltrials.gov ID: NCT03591965) and is in phase I trials for non-small-cell lung cancer (clinicaltrials.gov ID: NCT01545947). Of note, CC-223 has also reported sub-micromolar activity on Vascular Endothelial Growth Factor Receptor 3 (VEGR3, IC_50_ = 651.0 nM) [156], a target implicated in PC development [162]. Moreover, Onatasertib has also been found to inhibit PC-3 proliferation both in vitro and in vivo [124]. Interestingly, Onatasertib has also been found to exhibit antitumor effects in a murine xenograft model of glioblastoma multiforme (U87MG cells) [124], demonstrating the ability to cross the blood–brain barrier. Considering the reported multi-target activity profile, the results observed in the in vitro and in vivo data, and the tolerability observed in patients at the clinical trials [163,164,165], Onatasertib can be considered a valuable candidate for fast repurposing against PC, especially for patients that present metastases spread at different sites.

**VS-5584 (DB12986)** is a potent and selective dual inhibitor of PI3K/mTOR, with potential antineoplastic activity. In particular, VS-5584 has reported low nanomolar activity against *α*, β, γ, and δ PI3K isoforms (IC_50_ = 16 nM, IC_50_ = 68 nM, IC_50_ = 25 nM, IC_50_ = 42 nM, respectively) [126]. Moreover, it was demonstrated to inhibit mTOR low in the low nanomolar range (IC_50_ = 37 nM), and PC-3 cells in in vitro (IC_50_ = 180 nM) and in vivo experiments [125,126]. Although phase I clinical trials of VS-5584 against mesothelioma (clinicaltrials.gov ID: NCT02372227) and non-hematological malignancies or lymphoma (clinicaltrials.gov ID: NCT01991938) have been terminated due to study de-prioritization issues, the activity profiles and good tolerability of VS-5584 make it an optimal candidate for PC treatment [125,126].

**BIIB021 (DB12359)** is a potent, orally active inhibitor of Heat Shock Protein 90 (Hsp90) with anticancer activity [157,158,166]; BIIB021 has been demonstrated to inhibit both Hsp90 isoforms α and β (IC_50_ = 5.1 nM, IC_50_ = 17 nM, respectively) [167,168]. The ability of this compound to inhibit Hsp90 is of primary interest for prostate cancer treatment, considering the role of this target in PC development and progression [50]. Moreover, this compound also inhibits the mitochondrial isoform TRAP1 (Hsp75kDa, K_i_ = 62 nM), which is often upregulated in prostate cancer [169,170]. Interestingly, BIIB021 has been extensively evaluated in clinical trials for breast cancer (phase II, clinicaltrials.gov ID: NCT01004081) and solid tumors (phase I, clinicaltrials.gov ID: NCT00618735). BIIB021 showed also growth inhibition activity on PC-3 cells in the nanomolar range in vitro and tumor growth inhibition on CW22R xenografts in vivo [127,128]. Taking into account its multi-target activity profile and the potent antiproliferative effects on different PC cell models, BIIB021 can be considered another promising candidate for fast repurposing against prostate cancer. 

**Adapalene (CD-271, DB00210)** is a synthetic retinoid that activates the retinoic acid receptor gamma subtype (RARγ), which is currently approved for topical treatment of *Acne Vulgaris* [171]. Adapalene exhibits K_i_ = 130 nM against RARy, and K_i_ = 1100 nM and K_i_ = 34 nM against RARα and RARβ, respectively [172]. Moreover, retinoic acid receptors (RARs) are known to play a role in cell differentiation and tumor suppression; therefore, they could represent relevant targets for the treatment of prostate cancer [173,174,175,176,177]. According to recent research data, Adapalene exerts strong in vitro and in vivo antiproliferative effects on different PC cell lines (i.e., DU-145, PC-3, RM-1), by inducing DNA damage, S-phase cell cycle arrest, and apoptosis [129,130]. One of the major limitations of Adapalene is the poor water solubility, which hampers in vivo bioavailability, with this effect being common to retinoids. However, recent studies have demonstrated that advanced delivery systems can significantly improve distribution, delivery, and efficacy of insoluble drugs, such as Adapalene [178], making them valuable therapeutics also for systemic treatment. On these premises, Adapalene can be a valuable candidate for fast repurposing against PC.

**Picropodophyllin (AXL1717, PPP, DB12802)** is a potent inhibitor of the insulin-like growth factor 1 receptor (IGF1R), which is overexpressed in a variety of human cancers, and it plays a critical role in the growth and survival of many types of tumors [179]. PPP is the *cis*-isomer of podophyllotoxin (PPT), which retains the antineoplastic activity of PPT, but without the toxicity derived from the β-tubulin binding and the modulation of DNA Topoisomerase II [180,181]. According to literature data, PPP potently suppresses tumor cell proliferation and induction of apoptosis in several types of tumor cells [182,183,184,185]. Moreover, a recent study has also demonstrated that PPP can induce antiproliferative effects, promoting cell cycle arrest and apoptosis on LNCaP and DU-145 cells, through the production of ROS species and the inhibition of the PI3K/AKT signaling pathway [131]. PPP was also tested in vitro and in vivo on PC-3 cells and other IGF1R-bearing cancerous cells, demonstrating good antiproliferative and tumor-inhibiting effects [133]. Moreover, Picropodophyllin showed also good BBB permeability in intracerebral xenograft models of glioblastoma [186], suggesting potential efficacy against PC metastases in the brain. PPP has completed phase II trials on squamous cell carcinoma (clinicaltrials.gov ID: NCT01561456) and non-small-cell lung cancer (clinicaltrials.gov ID: NCT01466647), demonstrating good tolerability. The observed in vitro and in vivo efficacy, in combination with the already established safety profile, make PPP an attractive candidate for fast repurposing against prostate cancer.

**VS-4718 (DB15273)** is an inhibitor of Focal Adhesion Kinase (FAK, IC_50_ = 1.5 nM) [134], which is a target often overexpressed in PC, and it is involved in the regulation activation of several major tumorigenic pathways behind pathological growth and survival of prostate cancer [187]. While phase I trials of VS-4718 against metastatic non-hematologic malignancies (clinicaltrials.gov ID: NCT01849744) and advanced pancreatic cancer (clinicaltrials.gov ID: NCT02651727) have been discontinued due to study de-prioritization issues, it remains a valuable repurposing candidate for prostate cancer treatment, according to its antiproliferative activity on PC-3 cells and the low nanomolar activity on the FAK kinase.

**BMS-214662 (DB12234)** is a potent inhibitor of the enzyme farnesyltransferase, which is a target involved in the post-translational regulation of several proteins involved in signal transduction [188]. According to recent data, BMS-214662 presents low micromolar antiproliferative activity on PC-3 and LNCaP cells [135]. Results of phase I trials of this compound against acute promyelocytic leukemia (clinicaltrials.gov ID: NCT00006213) and adult solid tumors in combination with Paclitaxel (clinicaltrials.gov ID: NCT00006018) including 2 PC patients [136] suggest that further clinical assessments on BMS-214662, either as a single agent or as a combination of drugs, could support its repurposing on PC.

Another valuable candidate is **Flubendazole (DB08974)**, which was demonstrated to potently inhibit the proliferation of CRPC cells in different in vitro and in vivo models [137]. This compound acts by inducing cell cycle arrest in the G2/M phase while promoting ferroptosis in CRPC cells [137]. In PC-3 and DU-145, a cell viability decrease was observed after treatment with 0.1 µM of Flubendazole [137]. Moreover, it also strongly upregulates the expression of p53 and its downstream effector p21, which are genes frequently mutated in several types of cancer [189]. Of note, Flubendazole has been found to exert synergistic effects with 5-fluorouracil against CRPC in PC-3 xenograft models [137], and it has been reported to exert anti-tumor effects on different types of cancers [190]. Notably, this compound is closely related to mebendazole, another anthelmintic drug that has recently been repurposed in combination with docetaxel against prostate cancer [191]. On these premises, Flubendazole is worth further assessments for potential repurposing against PC.

Another broad-spectrum anthelminthic agent potentially repositionable against PC is **Albendazole (DB00518)**, which showed potent antiproliferative effects toward PC-3 and DU-145 at 0.1 µM concentration and 0.5 µM against LNCaP [138]. Unfortunately, recent studies showed that high doses of Albendazole for a prolonged time could result in toxicity according to results of the phase I trial of this compound including 2 PC patients, suggesting frequent dose monitoring [139]. However, the fact that this compound presents potent PC antiproliferative activity and passed all phases of clinical trials as an antihelmintic drug makes it very interesting for repositioning against prostate cancer.

## 3. Materials and Methods

### 3.1. Curation of the Ligands and Targets Dataset

Bioactivity records of molecules assayed on RWPE1 (androgen responsive adult b prostatic epithelial cells) and malignant prostate cell lines PC-3, DU-145, LNCaP, LNCaP clone FGC, Vcap, 22Rv-1, LAPC4, and PWR1E were first acquired from the ChEMBL database (https://www.ebi.ac.uk/chembl/, accessed on 1 November 2021) [25,192]. Then, the molecules were filtered to retain only those with bioactivity data expressed by means of: (i) *“*Standard Type*”* equal to GI_50_, EC_50_, or IC_50_; (ii) “Standard Relation” equal to “<”, “>”, or “=”; (iii) “Standard Unit” in the nanomolar range (i.e., “nM”). For each ligand, duplicate records deriving from multiple assays conducted under different experimental conditions were removed, retaining the one with the best activity value (i.e., 54871 unique compounds). Finally, only ChEMBL compounds that showed an activity below 1 µM on at least one PC cell line (herein considered as “highly active”) and a molecular weight in the range of 180–850 Da were retained for the subsequent calculations. The performed filtration resulted in 6626 compounds, each of them with reported activity data on at least one PC cell line. 

Compounds reported in the DrugBank database (https://go.drugbank.com/, accessed on 1 November 2021) [24] were downloaded (i.e.,11912 ligands) and filtered to remove those already present in the curated dataset of ChEMBL ligands. The two datasets were compared by means of their molecular properties (i.e., LogP, polar surface area (PSA), number of hydrogen bond donors (HBD) and acceptors (HBA), molecular weight (MW), volume, and ovality), calculated by using the “RDKit Descriptor Calculation” and “CDK Molecular properties” *KNIME* nodes [193]. Then, the curated datasets of ChEMBL and DrugBank ligands were prepared for the similarity assessments, as follows. Canonical smiles were first generated for all the compounds. Then, hydrogen atoms were added to the structures of the compounds and salt counterions were removed. This phase of the ligands preparation was performed with the OpenEye Python toolkits [194]. The preparation of the compounds for the 3D similarity estimations required also a further step of conformational sampling, which was performed with the OMEGA2 software, adopting the same parameters used in our previous study [195,196].

### 3.2. Ligand-Based Calculations

The similarity degree of the ligands reported in the curated ChEMBL and DrugBank datasets were evaluated as follows. First, an *all-against-all* 2D-fingerprint-based similarity assessment was performed by using an *in-house*-developed Python script. The degree of 2D similarity was calculated according to the MACCS and circular (i.e., ECFP4) type of fingerprints available on OpenEye Python toolkits [194], and by using the Tanimoto coefficient (Tc) as an index for ligands similarity measurement. Then, the results of the 2D similarity calculations were filtered to retain only those with MACCS and ECFP4 Tanimoto coefficients equal to or higher than 0.8 and 0.3, respectively, in agreement with previously reported studies [196]. Afterward, extensive 3D shape and atom type similarity calculations of compounds resulting as significantly similar in the 2D estimations were performed by using the ROCS software [117]. In this case, the similarity degree between ligands was assessed according to the Tanimoto Combo (TCc) coefficient [117]. Finally, the results of the 3D-shape-based similarity calculations were filtered to retain only similarity records with a TCc ≥ 1.5, which is a commonly accepted threshold of similarity [196].

### 3.3. Data Integration

The results obtained in the similarity estimations were associated with information on targets and PC-cell-based bioactivity data and filtered to retain only DrugBank ligands that resulted as significantly similar to ChEMBL compounds with potent antiproliferative activity against prostate cancer cells. Our investigations focused on compounds that already possessed annotations on prostate cancer according to literature data, which are more likely to be assessed in clinical trials and potentially repurposed against PC. In particular, data related to trials on PC or solid tumors including patients with prostate cancer were retrieved from the clinicaltrials.gov website (https://clinicaltrials.gov/, accessed on 1 November 2021) [29]. In addition, bioactivity records on molecular targets reported on ChEMBL were also associated with the DrugBank ligands that emerged from the similarity estimations. In this phase, DrugBank ligands were associated with activity data deriving from target-based experiments on human proteins and expressed by means of the IC_50_, K_i_, K_d_, or EC_50_ types. Duplicate records reported for the same targets were removed, retaining the best value, and the resulting activity data were classified as “highly active” (IC_50_, K_i_, K_d_, or EC_50_ ≤ 1 µM), “scarcely active” (1 µM ≥ IC_50_, K_i_, K_d_, or EC_50_ ≤ 10 µM), and “inactive” (IC_50_, K_i_, K_d_, or EC_50_ ≥ 10 µM). All steps of data curation were performed with the Pandas Python library [197].

### 3.4. Analysis of Biological Target Activity Annotations 

An analysis of the activity annotations on biological targets associated with ChEMBL compounds with known antiproliferative records on PC-3, DU-145, and LNCaP PC cancer cell lines (54773 unique molecules) was also carried out. In particular, bioactivity data related to these compounds were first retrieved from ChEMBL. Then, these records were filtered to retain only those with bioactivity data expressed by means of: (i) “Standard Type” equal to IC_50_, K_i_, K_d_, or EC_50_; (ii) “Standard Relation” equal to “=”; (iii) “Standard Unit” in the nanomolar range (i.e., “nM”); (iv) “Target type” equal to “Single protein”; (v) “Target Organism” equal to “Homo Sapiens”; vi) “Assay Type” equal to “B” (i.e., binding assay type). Duplicate records reported for the same target were removed, retaining the best value. Afterward, only targets with at least 10 bioactivity annotations were retained. The correlation between the activity on the target and antiproliferative activity on PC cells was assessed with *Spearman’s Rho (ρ_s_)* correlation coefficient, which was calculated with the Scipy Python library [198]. A *p*-value equal to or lower than 0.05 was considered statistically significant for the analyses. Biological targets with *ρ_s_* ≥ 0.40 were finally retained [199]. 

## 4. Conclusions

In this study, an integrated in silico workflow based on extensive similarity estimations, and data integration and analysis were devised in the search for compounds with favorable safety and tolerability profiles for repurposing against PC therapy. In particular, the candidates were first selected according to their similarity degree with respect to potent molecules with reported potent antiproliferative activity against PC cells (below 1 µM). 

Moreover, an analysis of the activity annotations reported for compounds also allowed us to identify biological targets whose modulation presents a significant correlation with known antiproliferative activities reported on different PC cell lines (see Table 1 and Figure 2). Of note, some of the identified biological target-cell activity associations have previously been reported in the literature, while others have never been described, potentially providing clues for future drug discovery efforts against PC.

Then, extensive integration and analyses on compounds annotations reported in different databases and literature searches allowed us to select 10 particularly interesting candidates for fast repurposing against prostate cancer (see Table 2). The identified candidates have already been assessed in clinical trials for other pathologies, and most of them showed good safety and tolerability profiles when tested on patients. Remarkably, they have potent in vitro and in vivo activity on PC cells, as well as activity on targets relevant to PC. Some of the identified compounds have demonstrated to be able to cross the BBB, thus being potentially useful for the treatment of forms of prostate cancer that metastasize to neuronal tissues. These compounds represent ideal candidates for fast enrollment in clinical trials against PC, either alone or in combination with other drugs. 

## Figures and Tables

**Table 1:** Targets identified from the analyses performed in the CHEMBL dataset, whose inhibitory activity data correlate with the activity on PC cells. The table reports only targets whose lower confidence interval of *Spearman’s Rho (ρ_s_)* resulted as equal or higher than 0.4 (*ρ_s_* ≥ 0.4, *p*-value < 0.001).

Target Name	Protein Family	UniProt Name *	PC Cell
Kinesine-like protein KIF11	Kinesin family, BimC subfamily	KIF11	PC-3
Heat shock protein HSP 90-alpha	Heat shock protein 90 family	HS90A	PC-3
Histone deacetylase 4	Histone deacetylase family, HD type 2 subfamily	HDAC4	PC-3
DNA Topoisomerase I	Type IB topoisomerase family	TOP1	PC-3
Serine/threonine-protein kinase mTOR	PI3/PI4-kinase family	MTOR	PC-3
Histone deacetylase 1	Histone deacetylase family, HD type 1 subfamily	HDAC1	PC-3
PI3-kinase p110-gamma subunit	PI3/PI4-kinase family	PK3CG	PC-3
Voltage-gated T-type calcium channel alpha 1-G subunit	Calcium channel alpha-1 subunit family, CACNA1G subfamily	CAC1G	DU-145
Cyclin-dependent kinase 1	CMGC Ser/Thr protein kinase family, CDC2/CDKX subfamily	CDK1	DU-145

Note: * The complete UniProt Name of the targets includes also the suffix “_HUMAN”.

**Table 2:** Compounds identified from the similarity screenings and relevant association to PC, proposed as potential repurposing candidates. The first 5 compounds underlined in the table have already completed or are currently enrolled in clinical trials against PC. “Max Phase” was obtained from ChEMBL. “Disease” and “Primary target” were obtained from TTD. “Primary target” also contains the target UniProt name and the molecule’s mechanism of activity on the target in square brackets.

Molecule Name	2D Structure	Activity In Vitro	Activity In Vivo	Max Phase	Clinical Trials	Disease	Primary Target *
**Azacitidine**	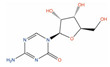	Cell viability ~85% (0.5 µM, PC-3), ~80% (0.1 µM, 22Rv1) [118]		4	NCT00503984; Phase I [119]	Myelodysplastic syndrome (Approved)	RNA polymerase II [Inhibitor], RNA methyltransferase [Inhibitor]
**Trimetrexate**	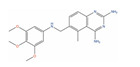			4	Phase II (Advanced hormone refractory-PC) [120]	Toxoplasmosis (Approved)	Dihydrofolate reductase (DYR) [Inhibitor]
**Afuresertib**	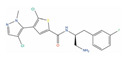	IC50 = 104 nM (LNCaP) [121]		2	NCT04060394	Leukemia (Phase 2); Solid tumor/cancer (Phase 2); Multiple myeloma (Phase 1)	RAC-alpha serine/threonine-protein kinase (AKT1) [Modulator]
**Vistusertib**	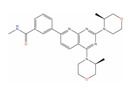		Tumor reduction from 15 mg/kg (PC-3) [122]	2	NCT02064608; NCT01884285	Solid tumor/cancer (Phase 2)	Serine/threonine-protein kinase mTOR (mTOR) [Inhibitor]; Mammalian target of rapamycin complex 1 (mTORC1) [Inhibitor]; Target of rapamycin complex 2 MAPKAP1 (MTORC2) [Inhibitor]
**Exatecan**	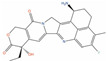			3	NCT00004045	Solid tumor/cancer (Phase 3)	DNA topoisomerase I (TOP1) [Inhibitor]
**Bimiralisib**	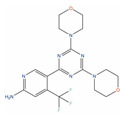		T/C = 31–12% with 5–15 mg/kg (PC-3) [123]	2		Squamous head and neck cell carcinoma (Phase 2); Pain (Phase 1)	PI3-kinase beta (PIK3CB) [Inhibitor]; Serine/threonine-protein kinase mTOR (mTOR) [Inhibitor]; PI3-kinase gamma (PIK3CG) [Inhibitor]
**Onatasertib** **(CC-223)**	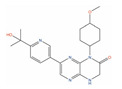	IC50 = 0.114 µM (PC-3)	Tumor reduction from 5 mg/kg (PC-3) [124]	2		Solid tumor/cancer (Phase 1/2)	Serine/threonine-protein kinase mTOR (mTOR) [Modulator]
**VS-5584**	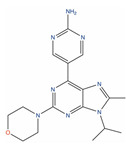	IC50 = 0.18 µM (PC-3) [125]	TCI = 79% at 25 mg/kg (PC-3) [126]	1		Solid tumor/cancer (Phase 1); Malignant Mesothelioma (Phase 1)	Serine/threonine-protein kinase mTOR (mTOR) [Modulator]; PI3-kinase gamma (PIK3CG) [Modulator]; Mammalian target of rapamycin complex 1 (mTORC1) [Inhibitor]; Target of rapamycin complex 2 MAPKAP1 (MTORC2) [Inhibitor]
**BIIB021**	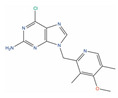	GI50 = 0.28 µM (PC-3) [127]	Tumor growth inhibition 87% at 120 mg/kg (CWR22) [128]	2		Breast cancer (Phase 2)	Heat shock protein 90 alpha (HSP90A) [Inhibitor]
**Adapalene**	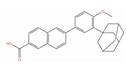	IC50 = 8.23 µM at 24 h (RM-1); Cell viability (0.01 µM, 72 h)~65% (DU-145) [129,130]	Tumor reduction from 30 mg/kg (RM-1) [131]	4		Acne vulgaris (Approved)	Retinoic acid receptor gamma (RARG) [Agonist]
**Picropodophyllin**	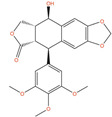	IC50 = 0.802 µM (DU-145); IC50 = 0.899 µM (LNCaP) [132]; IC50 = 100 nM (PC-3) [133]	Tumor reduction from 20 mg/kg (PC-3) [133]	2		Solid tumor/cancer (Phase 2)	Insulin-like growth factor I receptor (IGF1R) [Inhibitor]
**VS-4718**	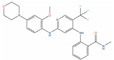	IC50 = 0.62 µM (DU-145) [134]		1		Solid tumour/cancer (Phase 1)	Focal adhesion kinase 1 (FAK) [Inhibitor]
**BMS-214662**	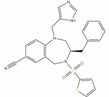	IC50 = 0.16 µM (PC-3); IC50 = 0.14 µM (LNCaP) [135]		1	Phase I combination with placitaxel on 2 PC patients [136]	Non-small-cell lung cancer (Phase 1)	Farnesyl protein transferase (Ftase) [Modulator]
**Flubendazole**	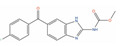	Cell viability (0.1 µM, 72 h) ~65% (PC-3), ~80% (DU-145) [137]	Tumor reduction from 10 mg/kg (PC-3). [137]	4		Worm infection (Approved)	
**Albendazole**	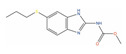	Cell viability (48 h) ~70% (0.1 µM; PC-3, DU-145); ~90% (0.5 µM, LNCaP) [138]		4	Phase I 2 PC patients [139]	Worm infection (Approved)	

Note: * The complete UniProt Name of the targets includes also the suffix “_HUMAN”. “Clinical Trials” contains clinical trials on PC or on solid tumors, when PC patients were enrolled.

## Data Availability

All relevant data presented in this study is available within the article or supplementary material. Further inquiries can be provided by the authors upon request.

## Supplementary Materials

The following supporting information can be downloaded at: https://www.mdpi.com/article/10.3390/ijms24043135/s1.
