# Supplementary material for: Identification of Promising Drug Candidates against Prostate Cancer through Computationally-Driven Drug Repurposing
Source: Int J Mol Sci. 2023 Feb 5;24(4):3135. doi: 10.3390/ijms24043135 (PMC9964599; doi:10.3390/ijms24043135)
Supplement: Supplementary file 1 [file ijms-24-03135-s001.zip › ijms-2160612-supplementary.pdf]

"Identification of promising drug candidates against prostate cancer through computationally-driven drug repurposing"

Leonardo Bernal,[1,2] Luca Pinzi,[1] Giulio Rastelli[1]*

[1] Department of Life Sciences, University of Modena and Reggio Emilia, Via Giuseppe Campi 103, 41125 Modena, Italy.
[2] Clinical and Experimental Medicine PhD Program, University of Modena and Reggio Emilia, Modena, Italy.

*** Correspondence to**: Prof. Giulio Rastelli, Department of Life Sciences, University of Modena and Reggio Emilia, Via Giuseppe Campi 103, 41125 Modena, Italy. Tel +39 059 2058564, Email giulio.rastelli@unimore.it

Table of contents – supporting information

Tables
Table S1 …………………………………………………………………………..…………… 3
Table S2 ……………………………………………………………………….……….………… 4
Table S3 …..…………………………………………………………………………..…...…… 5
Table S4 ………………………………………………………………………………………… 7
Table S5 ….……………………………………………………………………………......…. 9
Table S6 ….………………………………………………………………………………... 14
Table S7 …………………………………………………………………………….……….. 16
Table S8 ……………………………………………..…………………………….….………. 22

Figures
Figure S1 ………………………………………………………………………….………………. 23
Figure S2 ….…………………………………………………………………………………..... 24
Figure S3 ….……………………………………………………………………….…………..... 25
Figure S4 ….……………………………………………………………………………….…..... 26
Figure S5 ….……………………………………………………………………………..…...... 27

Table S1. Number of molecules with activity records on PC cell lines retrieved from ChEMBL, before and after the filtration process.

	PC-3	*DU-145*	*LNCaP*	*Vcap*	*LNCaP clone FGC*	*22Rv1*	*LAPC4*	*PWR-1E*	*RWPE-1*
Starting dataset	***67207***	***52325***	***10159***	***785***	***192***	***122***	***300***	***39***	***474***
IC_{50}	*15973*	*8882*	*4314*	*372*	*164*	*112*	*35*	*22*	*353*
EC_{50}	*558*	*223*	*215*	*0*	*8*	*0*	*0*	*0*	*0*
GI_{50}	*32173*	*33696*	*342*	*41*	*2*	*0*	*7*	*0*	*32*
After filtration	***44014***	***40387***	***3610***	***377***	***140***	***91***	***26***	***16***	***2***
IC_{50}	*12437*	*7711*	*3195*	*340*	*140*	*91*	*19*	*16*	*2*
EC_{50}	*404*	*177*	*100*	*0*	*0*	*0*	*0*	*0*	*0*
GI_{50}	*31173*	*32499*	*315*	*37*	*0*	*0*	*7*	*0*	*0*

Table S2. Number of molecules tested on PC cells, classified according to the activity type and value. In particular, the compounds were classified as: i) "*highly active*" (HA) when their activity values is lower or equal to 1 μM; ii) "*scarcely active*" molecules (SA) when their activity values is higher than 1 μM, and lower or equal to 10 μM, and; iii) inactive molecules (INA) when their activity values is higher than 10 μM.

	IC_{50}			***EC_{50}***			***GI_{50}***		
	HA	***SA***	***INA***	***HA***	***SA***	***INA***	***HA***	***SA***	***INA***
PC-3	*2083*	*3703*	*6651*	*148*	*124*	*132*	*2626*	*5891*	*22656*
DU-145	*1202*	*2352*	*4157*	*18*	*62*	*97*	*3224*	*6317*	*22958*
LNCaP	*956*	*1060*	*1179*	*11*	*24*	*65*	*102*	*121*	*92*
Vcap	*46*	*118*	*176*	*0*	*0*	*0*	*10*	*3*	*24*
LNCaPclone FGC	*50*	*35*	*55*	*0*	*0*	*0*	*0*	*0*	*0*
22Rv1	*25*	*28*	*38*	*0*	*0*	*0*	*0*	*0*	*0*
LAPC4	*10*	*8*	*1*	*0*	*0*	*0*	*0*	*0*	*7*
PWR-1E	*15*	*1*	*0*	*0*	*0*	*0*	*0*	*0*	*0*
RWPE-1	*1*	*1*	*0*	*0*	*0*	*0*	*0*	*0*	*0*

Table S3. All targets that emerged from the analysis of activity annotations of ligands with PC-3, DU-145, and LNCaP cell-based activity data reported in ChEMBL. The targets with significant Spearman's Rho coefficient of correlation ($\rho_s \geq 0.4$, p-value < 0.001) are highlighted in bold.

Target Name	Protein family	Target ChEMBL ID	Uniprot ID *	Cell lines
Serine/threonine-protein kinase PIM1	Protein kinase superfamily, CAMK Ser/Thr protein kinase family, PIM subfamily	CHEMBL2147	PIM1	PC-3
PI3-kinase p110-alpha subunit	PI3/PI4-kinase family	CHEMBL4005	PK3CA	PC-3
Tyrosine-protein kinase LCK	Protein kinase superfamily, Tyr protein kinase family, SRC subfamily	CHEMBL258	LCK	PC-3
Glycogen synthase kinase-3 beta	Protein kinase superfamily, CMGC Ser/Thr protein kinase family, GSK-3 subfamily	CHEMBL262	GSK3B	PC-3
Cyclin-dependent kinase 2	Protein kinase superfamily, CMGC Ser/Thr protein kinase family, CDC2/CDKX subfamily	CHEMBL301	CDK2	PC-3; DU-145
**Cyclin-dependent kinase 1**	**Protein kinase superfamily, CMGC Ser/Thr protein kinase family,CDC2/CDKX subfamily**	**CHEMBL308**	**CDK1**	**PC-3; DU-145**
Serine/threonine-protein kinase AKT2	Protein kinase superfamily, AGC Ser/Thr protein kinase family, RAC subfamily	CHEMBL2431	AKT2	PC-3
Serine/threonine-protein kinase TAO1	Protein kinase superfamily, STE Ser/Thr protein kinase family, STE20 subfamily	CHEMBL5261	TAOK1	PC-3
Serine/threonine-protein kinase D2	Protein kinase superfamily, CAMK Ser/Thr protein kinase family, PKD subfamily	CHEMBL4900	KPCD2	PC-3
Dual specificity mitogen-activated protein kinase kinase 5	Protein kinase superfamily, STE Ser/Thr protein kinase family, MAP kinase kinase subfamily	CHEMBL4948	MP2K5	PC-3
PI3-kinase p110-delta subunit	PI3/PI4-kinase family	CHEMBL3130	PK3CD	PC-3
PI3-kinase p110-beta subunit	PI3/PI4-kinase family	CHEMBL3145	PK3CB	PC-3
**PI3-kinase p110-gamma subunit**	**PI3/PI4-kinase family**	**CHEMBL3267**	**PK3CG**	**PC-3**
**Serine/threonine-protein kinase mTOR**	**PI3/PI4-kinase family**	**CHEMBL2842**	**MTOR**	**PC-3**
MAP kinase p38 alpha	Protein kinase superfamily, CMGC Ser/Thr protein kinase family, MAP kinase subfamily	CHEMBL260	MK14	PC-3
**Histone deacetylase 1**	**Histone deacetylase family, HD type 1 subfamily**	**CHEMBL325**	**HDAC1**	**PC-3; DU-145; LNCaP**
Histone deacetylase 10	Histone deacetylase family, HD type 2 subfamily	CHEMBL5103	HDA10	PC-3
Histone deacetylase 8	Histone deacetylase family, HD type 1 subfamily	CHEMBL3192	HDAC8	PC-3
**Histone deacetylase 4**	**Histone deacetylase family, HD type 2 subfamily**	**CHEMBL3524**	**HDAC4**	**PC-3**
Histone deacetylase 5	Histone deacetylase family, HD type 2 subfamily	CHEMBL2563	HDAC5	PC-3
DNA topoisomerase II alpha	Type II topoisomerase family	CHEMBL1806	TOP2A	PC-3; DU-145
Proteasome Macropain subunit MB1	Peptidase T1B family	CHEMBL4662	PSB5	PC-3
Proteasome Macropain subunit	Peptidase T1B family	CHEMBL3492	PSB2	PC-3
Histone deacetylase 2	Histone deacetylase family, HD type 1 subfamily	CHEMBL1937	HDAC2	PC-3
Histone deacetylase 11	Histone deacetylase family	CHEMBL3310	HDA11	PC-3
Dihydrofolate reductase	Dihydrofolate reductase family	CHEMBL202	DYR	PC-3; DU-145
Androgen Receptor	Nuclear hormone receptor family, NR3 subfamily	CHEMBL1871	ANDR	PC-3
DNA topoisomerase I	Type IB topoisomerase family	CHEMBL1781	TOP1	PC-3
**Heat shock protein HSP 90-alpha**	**Heat shock protein 90 family**	**CHEMBL3880**	**HS90A**	**PC-3**
Alpha-1b adrenergic receptor	G-protein coupled receptor 1 family, Adrenergic receptor subfamily, ADRA1B sub-subfamily	CHEMBL232	ADA1B	PC-3
**Kinesin-like protein 11**	**TRAFAC class myosin-kinesin ATPase superfamily, Kinesin family, BimC subfamily**	**CHEMBL4581**	**KIF11**	**PC-3**
Nicotinamide phosphoribosyltransferase	NAPRTase family	CHEMBL1744525	NAMPT	PC-3; DU-145
Fatty acid synthase		CHEMBL4158	FAS	PC-3
**Voltage-gated T-type calcium channel alpha-1G subunit**	**Calcium channel alpha-1 subunit (TC 1.A.1.11) family, CACNA1G subfamily**	**CHEMBL4641**	**CAC1G**	**DU-145**
Citron Rho-interacting kinase	Protein kinase superfamily, AGC Ser/Thr protein kinase family	CHEMBL5579	CTRO	DU-145
Signal transducer and activator of transcription 3	Transcription factor STAT family	CHEMBL4026	STAT3	DU-145
Serine/threonine-protein kinase RIPK2	Protein kinase superfamily, TKL Ser/Thr protein kinase family	CHEMBL5014	RIPK2	DU-145
Vascular endothelial growth factor receptor 2	Protein kinase superfamily, Tyr protein kinase family, CSF-1/PDGF receptor subfamily	CHEMBL279	VGFR2	DU-145

1-acylglycerol-3-phosphate O-acyltransferase beta	1-acyl-sn-glycerol-3-phosphate acyltransferase family	CHEMBL4772	PLCB	DU-145
Epidermal growth factor receptor erbB1	Protein kinase superfamily, Tyr protein kinase family, EGF receptor subfamily	CHEMBL203	EGFR	DU-145
Serine/threonine-protein kinase Aurora-A	Protein kinase superfamily, Ser/Thr protein kinase family, Aurora subfamily	CHEMBL4722	AURKA	LNCaP
Bromodomain-containing protein 4		CHEMBL1163125	BRD4	LNCaP

Note: * the complete UniProt name of proteins includes also the suffix "_HUMAN".

Table S4. Spearman's Rho correlation coefficient (ρ_s) with confidence interval (CI) of the targets that emerged as relevant from the analysis of activity annotations of ligands with PC-3, DU-145, and LNCaP data in ChEMBL. The targets with an acceptable Spearman's Rho coefficient ($\rho_s \geq 0.4$, p-value < 0.001) are highlighted in bold.

Target Name	*Uniprot Name **	*PC-3*					*DU-145*					*LNCaP*				
		ρ_s	*p-value*	*N° of records*	*Lower CI*	*Upper CI*	ρ_s	*p-value*	*N° of records*	*Lower CI*	*Upper CI*	ρ_s	*p-value*	*N° of records*	*Lower CI*	*Upper CI*
**Kinesin-like protein 1**	**KIF11**	**0,89**	**5,52E-08**	**21**	**0,72**	**0,96**										
**Heat shock protein HSP 90-alpha**	**HS90A**	**0,84**	**3,19E-04**	**13**	**0,48**	**0,96**										
MAP kinase p38 alpha	MK14	0,79	3,75E-03	11	0,28	0,95										
Dual specificity mitogen-activated protein kinase kinase 5	MP2K5	0,78	1,65E-03	13	0,34	0,94										
Proteasome Macropain subunit	PSB2	0,78	2,91E-03	12	0,29	0,94										
**Histone deacetylase 4**	**HDAC4**	**0,75**	**1,61E-07**	**35**	**0,54**	**0,88**										
Fatty acid synthase	FAS	0,73	1,12E-02	11	0,14	0,94										
**DNA topoisomerase I**	**TOP1**	**0,72**	**1,85E-08**	**45**	**0,53**	**0,85**										
Serine/threonine-protein kinase AKT2	AKT2	0,69	9,85E-04	19	0,31	0,88										
Dihydrofolate reductase	DYR	0,68	9,10E-04	20	0,30	0,87	0,72	2,19E-04	21	0,38	0,89					
**Cyclin-dependent kinase 1**	**CDK1**	**0,68**	**3,59E-04**	**23**	**0,34**	**0,86**	**0,80**	**1,36E-05**	**21**	**0,53**	**0,92**					
**Serine/threonine-protein kinase mTOR**	**MTOR**	**0,67**	**2,64E-30**	**222**	**0,58**	**0,74**										
Serine/threonine-protein kinase TAO1	TAOK1	0,66	2,60E-02	11	0,02	0,92										
**Histone deacetylase 1**	**HDAC1**	**0,65**	**1,70E-22**	**179**	**0,54**	**0,73**	**0,58**	**2,29E-06**	**57**	**0,36**	**0,74**	**0,79**	**6,10E-03**	**10**	**0,23**	**0,96**
Androgen Receptor	ANDR	0,64	5,21E-03	17	0,19	0,87										
Serine/threonine-protein kinase D2	KPCD2	0,62	4,26E-02	11	-0,05	0,90										
Proteasome Macropain subunit MB1	PSB5	0,62	7,73E-04	26	0,27	0,82										
Nicotinamide phosphoribosyltransferase	NAMPT	0,60	6,26E-05	38	0,33	0,78	0,57	1,78E-02	17	0,07	0,84					
Cyclin-dependent kinase 2	CDK2	0,56	9,78E-03	20	0,12	0,82	0,66	5,46E-03	16	0,19	0,88					
Histone deacetylase 2	HDAC2	0,55	7,44E-06	58	0,33	0,72										
**PI3-kinase p110-gamma subunit**	**PK3CG**	**0,55**	**2,48E-11**	**124**	**0,41**	**0,67**										
DNA topoisomerase II alpha	TOP2A	0,54	2,93E-02	16	0,02	0,83	0,50	7,98E-03	27	0,12	0,75					
Histone deacetylase 11	HDA11	0,52	1,39E-02	22	0,09	0,78										
PI3-kinase p110-beta subunit	PK3CB	0,51	2,72E-05	61	0,28	0,68										
Glycogen synthase kinase-3 beta	GSK3B	0,50	2,99E-03	33	0,17	0,73										
Alpha-1b adrenergic receptor	ADA1B	0,49	7,64E-03	29	0,12	0,73										
PI3-kinase p110-delta subunit	PK3CD	0,48	3,67E-04	52	0,22	0,67										
Histone deacetylase 5	HDAC5	0,47	2,40E-02	23	0,04	0,75										

PI3-kinase p110-alpha subunit	PK3CA	0,46	2,93E-12	203	0,34	0,57										
Tyrosine-protein kinase LCK	LCK	0,44	1,07E-02	33	0,09	0,69										
Histone deacetylase 10	HDA10	0,42	3,40E-02	26	0,01	0,70										
Histone deacetylase 8	HDAC8	0,37	4,99E-04	87	0,16	0,54										
**Voltage-gated T-type calcium channel alpha-1G subunit**	**CAC1G**						**0,93**	**3,97E-05**	**11**	**0,68**	**0,99**					
Citron Rho-interacting kinase	CTRO						0,64	4,79E-02	10	-0,08	0,92					
Signal transducer and activator of transcription 3	STAT3						0,56	1,62E-02	18	0,08	0,83					
Serine/threonine-protein kinase RIPK2	RIPK2						0,55	4,16E-02	14	-0,03	0,85					
Vascular endothelial growth factor receptor 2	VGFR2						0,45	1,17E-03	50	0,18	0,65					
1-acylglycerol-3-phosphate O-acyltransferase beta	PLCB						0,41	4,24E-02	25	-0,01	0,70					
Epidermal growth factor receptor erbB1	EGFR						0,39	1,05E-03	69	0,15	0,58					
Serine/threonine-protein kinase Aurora-A	AURKA						0,33	2,90E-02	43	0,02	0,58					
Bromodomain-containing protein 4	BRD4											0,77	8,88E-03	10	0,18	0,95

Note: * the complete UniProt Name of proteins includes also the suffix "_HUMAN".

Table S5. 138 DrugBank ligands identified from the similarity calculations together with their ChEMBL IDs.

DrugBank ID	ChEMBL ID	CAS Number	Name
DB00210	CHEMBL1265	106685-40-9	Adapalene
DB00406	CHEMBL459265	7438-46-2	Gentian violet cation
DB00432	CHEMBL1129	70-00-8	Trifluridine
DB00518	CHEMBL1483	54965-21-8	Albendazole
DB00555	CHEMBL741	84057-84-1	Lamotrigine
DB00928	CHEMBL1489	320-67-2	Azacitidine
DB01157	CHEMBL119	52128-35-5	Trimetrexate
DB01958	CHEMBL329985		5-[4-Tert-Butylphenylsulfanyl]-2,4-Quinazolinediamine
DB02026	CHEMBL104829		Furo[2,3d]Pyrimidine Antifolate
DB02104	CHEMBL50514		2,4-Diamino-5-Methyl-6-[(3,4,5-Trimethoxy-N-Methylanilino)Methyl]Pyrido[2,3-D]Pyrimidine
DB02152	CHEMBL281948	97161-97-2	K-252a
DB02240	CHEMBL1190232	64046-79-3	Quinacrine mustard
DB02256	CHEMBL353955	951-78-0	2'-Deoxyuridine
DB02281	CHEMBL471524	6742-12-7	Formycin
DB02282	CHEMBL277041	2457-80-9	5'-S-methyl-5'-thioadenosine
DB02390			5-Bromo-N[2-(Dimethylamino)Ethyl]-9-Aminoacridine-4-Carboxamide
DB02427	CHEMBL325434		2,4-Diamino-6-[N-(2',5'-Dimethoxybenzyl)-N-Methylamino]Quinazoline
DB02472			7,8-dihydroinosine
DB02583	CHEMBL32113	175354-76-4	N6-(2,5-Dimethoxy-Benzyl)-N6-Methyl-Pyrido[2,3-D]Pyrimidine-2,4,6-Triamine
DB02753		40093-99-0	Selenoinosine
DB02798			Alpha-Methylene Adenosine Monophosphate
DB02842	CHEMBL287038		9-amino-n-[3-(dimethylamino)propyl]acridine-4-carboxamide
DB02857	CHEMBL375655	118-00-3	Guanosine
DB02896	CHEMBL388931	342-69-8	Methylthioinosine
DB02933	CHEMBL551561		5'-Deoxy-5'-(Methylthio)-Tubercidin
DB03034	CHEMBL420937	100986-86-5	D-Levofloxacin
DB03060			Sri-9662

DB03068	CHEMBL504567	3690-10-6	Zebularine
DB03199			4-Methoxy-E-Rhodomycin T
DB03351	CHEMBL151958		Sri-9439
DB03480	CHEMBL220467		Brequinar Analog
DB03626	CHEMBL94277		5-Methoxy-1,2-Dimethyl-3-(Phenoxymethyl)Indole-4,7-Dione
DB03671	CHEMBL1153	1672-46-4	Digoxigenin
DB03716	CHEMBL1212979		5'-Fluoro-5'-Deoxyadenosine
DB03735	CHEMBL1234254		9-(2-Deoxy-Beta-D-Ribofuranosyl)-6-Methylpurine
DB03763		65358-15-8	5-methyl-2'-deoxypseudouridine
DB03765	CHEMBL582887	85-94-9	2'-cytidylic acid
DB03804	CHEMBL1231486		5-Bromothienyldeoxyuridine
DB03952			9-(6-deoxy-beta-D-allofuranosyl)-6-methylpurine
DB03986			6-methyl-formycin A
DB03987	CHEMBL36245		2,4-Diamino-6-[N-(3',5'-Dimethoxybenzyl)-N-Methylamino]Pyrido[2,3-D]Pyrimidine
DB04011			2'-(4-Dimethylaminophenyl)-5-(4-Methyl-1-Piperazinyl)-2,5'-Bi-Benzimidazole
DB04163	CHEMBL100239		5-Phenylsulfanyl-2,4-Quinazolinediamine
DB04306	CHEMBL83547		5-[(4-Methylphenyl)Sulfanyl]-2,4-Quinazolinediamine
DB04385	CHEMBL1231969		3-Deazacytidine
DB04440	CHEMBL1399702	550-33-4	Nebularine
DB04441	CHEMBL290077	146-78-1	2-Fluoroadenosine
DB04546	CHEMBL202701	6736-58-9	3-Deazaadenosine
DB04604	CHEMBL99203	24386-93-4	5-iodotubercidin
DB04616	CHEMBL1082738		TACRINE(8)-4-AMINOQUINOLINE
DB04662	CHEMBL1094304		OLOMOUCINE II
DB04944	CHEMBL1551724	2627-69-2	Acadesine
DB04954	CHEMBL392149	204512-90-3	Tecadenoson
DB05585	CHEMBL492399	827031-83-4	Verubulin
DB05616		238074-89-0	4'-Methylene-5,8,10-trideazaaminopterin
DB05706	CHEMBL3249110	628290-43-7	13-deoxydoxorubicin
DB06198	CHEMBL105318	25526-93-6	Alovudine

DB06263	CHEMBL1186894	110267-81-7	Amrubicin
DB06420		92689-49-1	Annamycin
DB06433	CHEMBL2105467	171176-43-5	Tezacitabine
DB06581	CHEMBL404519	174022-42-5	Bevirimat
DB06721	CHEMBL113051	292618-32-7	Gimatecan
DB06813	CHEMBL1201746	146464-95-1	Pralatrexate
DB06896	CHEMBL509101		1-(4-fluorophenyl)-N-[3-fluoro-4-(1H-pyrrolo[2,3-b]pyridin-4-yloxy)phenyl]-2-oxo-1,2-dihydropyridine-3-carboxamide
DB06961	CHEMBL398346		5-(5-chloro-2,4-dihydroxyphenyl)-N-ethyl-4-[4-(morpholin-4-ylmethyl)phenyl]isoxazole-3-carboxamide
DB07052	CHEMBL195660		5'-S-ethyl-5'-thioadenosine
DB07153	CHEMBL1230365		6-methyl-5-[3-methyl-3-(3,4,5-trimethoxyphenyl)but-1-yn-1-yl]pyrimidine-2,4-diamine
DB07226			N-[4-(2-CHLOROPHENYL)-1,3-DIOXO-1,2,3,6-TETRAHYDROPYRROLO[3,4-C]CARBAZOL-9-YL]FORMAMIDE
DB07309	CHEMBL212522		5-BROMO-2-{[(4-CHLOROPHENYL)SULFONYL]AMINO}BENZOIC ACID
DB07322	CHEMBL380394		2-[(PHENYLSULFONYL)AMINO]-5,6,7,8-TETRAHYDRONAPHTHALENE-1-CARBOXYLIC ACID
DB07423	CHEMBL125236	401900-40-1	Andarine
DB07574			2-MERCAPTO-N-[1,2,3,10-TETRAMETHOXY-9-OXO-5,6,7,9-TETRAHYDRO-BENZO[A]HEPTALEN-7-YL]ACETAMIDE
DB07577	CHEMBL22148	27653-49-2	2,4-Diamino-5-phenyl-6-ethylpyrimidine
DB07638	CHEMBL236718		(3AS,4R,9BR)-2,2-DIFLUORO-4-(4-HYDROXYPHENYL)-1,2,3,3A,4,9B-HEXAHYDROCYCLOPENTA[C]CHROMEN-8-OL
DB07664	CHEMBL261720	443798-47-8	K-00546
DB07678			(9ALPHA,13BETA,17BETA)-2-[(1Z)-BUT-1-EN-1-YL]ESTRA-1,3,5(10)-TRIENE-3,17-DIOL
DB07707			(9BETA,11ALPHA,13ALPHA,14BETA,17ALPHA)-11-(METHOXYMETHYL)ESTRA-1(10),2,4-TRIENE-3,17-DIOL
DB07769	CHEMBL124718		S-3-(4-FLUOROPHENOXY)-2-HYDROXY-2-METHYL-N-[4-NITRO-3-(TRIFLUOROMETHYL)PHENYL]PROPANAMIDE
DB07791			4-{[4-(1-CYCLOPROPYL-2-METHYL-1H-IMIDAZOL-5-YL)PYRIMIDIN-2-YL]AMINO}-N-METHYLBENZENESULFONAMIDE
DB07810	CHEMBL45068	60-82-2	Phloretin
DB07812			N-[(1S)-2-amino-1-phenylethyl]-5-(1H-pyrrolo[2,3-b]pyridin-4-yl)thiophene-2-carboxamide
DB07877	CHEMBL383189		8-(6-BROMO-BENZO[1,3]DIOXOL-5-YLSULFANYL)-9-(3-ISOPROPYLAMINO-PROPYL)-ADENINE
DB08141	CHEMBL1187319		4-{[(2,6-difluorophenyl)carbonyl]amino}-N-[(3S)-piperidin-3-yl]-1H-pyrazole-3-carboxamide
DB08362	CHEMBL230354		N-(3-(8-CYANO-4-(PHENYLAMINO)PYRAZOLO[1,5-A][1,3,5]TRIAZIN-2-YLAMINO)PHENYL)ACETAMIDE
DB08471	CHEMBL1235660		1-(thiophen-2-ylacetyl)-4-(3-thiophen-2-yl-1,2,4-oxadiazol-5-yl)piperidine
DB08473	CHEMBL375530	53-85-0	Dichlororibofuranosylbenzimidazole
DB08541			[(3S)-9-hydroxy-1-methyl-10-oxo-4,10-dihydro-3H-benzo[g]isochromen-3-yl]acetic acid

DB08737			(3AS,4R,9BR)-4-(4-HYDROXYPHENYL)-1,2,3,3A,4,9B-HEXAHYDROCYCLOPENTA[C]CHROMEN-9-OL
DB08878	CHEMBL376180	54-62-6	Aminopterin
DB08974	CHEMBL1454946	31430-15-6	Flubendazole
DB11279	CHEMBL1589793	18198-35-1	Brilliant green cation
DB11410	CHEMBL37161	43210-67-9	Fenbendazole
DB11472	CHEMBL1371412	61570-90-9	Tioxidazole
DB11491	CHEMBL37858	98105-99-8	Sarafloxacin
DB11562	CHEMBL417990	485-49-4	Bicuculline
DB11648	CHEMBL2219422	1047644-62-1	Afuresertib
DB11674	CHEMBL198877	531-95-3	Equol
DB11872	CHEMBL257662	219923-05-4	ZD-6126
DB11881	CHEMBL399583	747412-64-2	AUY922
DB11925	CHEMBL2336325	1009298-59-2	Vistusertib
DB11933	CHEMBL452867	7724-76-7	Riboprine
DB12156	CHEMBL305686	73-03-0	Cordycepin
DB12185	CHEMBL1614650	171335-80-1	Exatecan
DB12222	CHEMBL305666	149882-10-0	Lurtotecan
DB12234	CHEMBL351706	195987-41-8	BMS-214662
DB12350	CHEMBL2068971	156722-18-8	Rostafuroxin
DB12359	CHEMBL467399	848695-25-0	BIIB021
DB12459	CHEMBL2111084	256411-32-2	Belotecan
DB12570	CHEMBL3586404	1228013-30-6	CC-223
DB12586		38390-45-3	Anhydrovinblastine
DB12802	CHEMBL283120	477-47-4	Picropodophyllin
DB12843	CHEMBL1075789	465-16-7	Oleandrin
DB12901	CHEMBL272557	69123-90-6	Fiacitabine
DB12925	CHEMBL2103852	1000852-17-4	Crolibulin
DB12957	CHEMBL1076257	10356-76-0	5-fluoro-2'-deoxycytidine
DB12986	CHEMBL3393066	1246560-33-7	VS-5584
DB13011	CHEMBL77101	119422-08-1	Diethylhomospermine

DB13103		236095-29-7	GPX-150
DB13304	CHEMBL313302	68-76-8	Triaziquone
DB13318	CHEMBL312862	477-30-5	Demecolcine
DB13465	CHEMBL1788401	31431-43-3	Ciclobendazole
DB13611	CHEMBL152649	3689-76-7	Chlormidazole
DB13756	CHEMBL1075790	1182-87-2	Peruvoside
DB13776	CHEMBL2104340	611-53-0	Ibacitabine
DB13865	CHEMBL1697741	4914-30-1	Dehydroemetine
DB13921	CHEMBL438605	20724-73-6	2'-C-methylcytidine
DB14025	CHEMBL278255	105956-97-6	Clinafloxacin
DB14122	CHEMBL378104	19309-14-9	Dihydroxymethoxychalcone
DB14178	CHEMBL571700	97-74-5	Tetramethylthiuram monosulfide
DB14846	CHEMBL4084907	1225037-39-7	Bimiralisib
DB14930		287114-80-1	Alovudine F-18
DB14933	CHEMBL438497	1341-23-7	Nicotinamide riboside
DB15273	CHEMBL3040440	1061353-68-1	VS-4718
DB15590	CHEMBL330498	477-52-1	beta-Apopicropodophyllin
DB15653	CHEMBL517231	865363-93-5	Islatravir
DB16070	CHEMBL4297468	1629677-75-3	KZR-616
DB16071		184302-49-6	FF-10502
DB16103	CHEMBL79280	195533-53-0	Batabulin
DB16407	CHEMBL519846	1011529-10-4	Azvudine

Table S6. 48 DrugBank ligands that are more similar to molecules of the ChEMBL dataset. The table includes fingerprint values, activity value on PC cell lines of the most similar molecule, and total number of similarities identified.

**DrugBank ID**	**Most similar ChEMBL molecule**	**MACCS**	**ECFP4**	**Tanimoto Combo**	**Shape Tanimoto**	**Color Tanimoto**	**Most similar molecule with activity on PC-3**	**Most similar molecule with activity on LNCaP**	**Most similar molecule with activity on DU-145**	**N° of similar molecules**
**DB08878**	CHEMBL1982461	0,897	0,882	1,98	0,98	1	GI50 = 118.03 nM		GI50 = 10.0 nM	1
**DB13318**	CHEMBL425414	0,814	0,9	1,979	0,979	1	IC50 = 17.6 nM			6
**DB07638**	CHEMBL278703	0,885	0,704	1,948	0,951	0,997	EC50 = 0.66 nM			2
**DB00210**	CHEMBL1180	0,885	0,844	1,913	0,982	0,931	IC50 = 430.0 nM	IC50 = 320.0 nM	IC50 = 280.0 nM	2
**DB16071**	CHEMBL555748	0,912	1	1,898	0,978	0,92	IC50 = 580.0 nM			2
**DB13756**	CHEMBL501533	1	0,903	1,894	0,98	0,914	GI50 = 0.7244 nM		GI50 = 0.1 nM	4
**DB14846**	CHEMBL2017974	1	1	1,87	0,976	0,894			IC50 = 910.0 nM	1
**DB06721**	CHEMBL112769	0,984	0,886	1,849	0,982	0,867	IC50 = 15.0 nM			8
**DB13011**	CHEMBL551695	0,862	0,5	1,846	0,928	0,918			IC50 = 60.0 nM	3
**DB08974**	CHEMBL1981545	0,886	0,846	1,827	0,934	0,894	GI50 = 348.34 nM			2
**DB04441**	CHEMBL1750	0,917	1	1,825	0,958	0,867	GI50 = 63.0 nM		GI50 = 125.0 nM	14
**DB05585**	CHEMBL1275943	0,912	0,692	1,818	0,979	0,838	GI50 = 509.0 nM		GI50 = 269.0 nM	1
**DB16103**	CHEMBL446353	0,891	0,842	1,811	0,977	0,833	GI50 = 468.0 nM			2
**DB00555**	CHEMBL264373	0,879	0,667	1,793	0,91	0,883	GI50 = 78.52 nM			1
**DB11648**	CHEMBL3137336	0,811	0,9	1,789	0,964	0,825		IC50 = 70.0 nM		1
**DB11410**	CHEMBL1707859	0,837	0,679	1,753	0,918	0,835	GI50 = 91.41 nM		GI50 = 309.03 nM	2
**DB06263**	CHEMBL178	0,852	0,886	1,73	0,95	0,78	IC50 = 26.0 nM	IC50 = 100.0 nM	IC50 = 24.5 nM	2
**DB06420**	CHEMBL1992472	0,833	0,889	1,702	0,933	0,769	GI50 = 273.53 nM		GI50 = 29.44 nM	1
**DB12185**	CHEMBL1970399	0,877	0,705	1,7	0,913	0,786	GI50 = 21.38 nM		GI50 = 10.0 nM	11
**DB12925**	CHEMBL1403937	0,811	0,885	1,694	0,961	0,733	GI50 = 462.0 nM		GI50 = 637.0 nM	3
**DB11925**	CHEMBL4239712	0,932	0,889	1,679	0,909	0,77	IC50 = 110.0 nM			1
**DB00928**	CHEMBL2064455	0,8	0,552	1,676	0,93	0,746	IC50 = 630.0 nM			1
**DB01157**	CHEMBL1965053	0,878	0,955	1,667	0,883	0,784			GI50 = 717.79 nM	4
**DB03068**	CHEMBL888	0,852	0,571	1,657	0,944	0,713	IC50 = 2.6 nM	IC50 = 512.0 nM	IC50 = 3.5 nM	1
**DB14122**	CHEMBL268838	0,8	0,344	1,652	0,875	0,777			GI50 = 330.0 nM	1
**DB07810**	CHEMBL268838	1	0,652	1,642	0,893	0,75			GI50 = 330.0 nM	1
**DB15273**	CHEMBL3393272	0,846	0,722	1,64	0,925	0,716	IC50 = 270.0 nM			1
**DB12459**	CHEMBL276820	0,844	0,775	1,636	0,85	0,786			GI50 = 17.99 nM	11
**DB00518**	CHEMBL9514	0,816	0,679	1,629	0,929	0,701			IC50 = 250.0 nM	1
**DB12843**	CHEMBL510771	0,978	0,968	1,627	0,916	0,712	GI50 = 25.59 nM		GI50 = 17.58 nM	1
**DB12234**	CHEMBL1738728	0,853	0,806	1,599	0,882	0,718			IC50 = 70.0 nM	1
**DB11562**	CHEMBL4644492	0,938	0,794	1,598	0,884	0,714	IC50 = 1000.0 nM			1
**DB08473**	CHEMBL236070	0,82	0,594	1,587	0,788	0,8	IC50 = 900.0 nM			1
**DB02152**	CHEMBL603469	0,852	0,833	1,583	0,902	0,681				3
**DB07664**	CHEMBL191003	0,883	0,655	1,583	0,88	0,703	IC50 = 120.0 nM			4
**DB12802**	CHEMBL1411422	0,805	0,543	1,573	0,893	0,68	GI50 = 33.11 nM		GI50 = 25.64 nM	3
**DB02282**	CHEMBL1814776	0,847	0,636	1,571	0,908	0,664	GI50 = 11.0 nM			1

**DB12986**	CHEMBL2314287	0,902	0,447	1,561	0,874	0,686	IC50 = 120.0 nM		2
**DB11872**	CHEMBL1985898	0,812	0,9	1,556	0,94	0,616	GI50 = 25.06 nM	GI50 = 18.49 nM	1
**DB12156**	CHEMBL236070	0,82	0,514	1,55	0,844	0,705	IC50 = 900.0 nM		3
**DB06433**	CHEMBL555748	0,823	0,679	1,548	0,897	0,651	IC50 = 580.0 nM		1
**DB11674**	CHEMBL278703	0,846	0,56	1,532	0,873	0,659	EC50 = 0.66 nM		1
**DB04944**	CHEMBL4284518	0,85	0,342	1,522	0,893	0,629	GI50 = 808.0 nM	GI50 = 562.34 nM	3
**DB12222**	CHEMBL1979572	0,831	0,702	1,52	0,853	0,667	GI50 = 22.91 nM	GI50 = 10.0 nM	1
**DB06813**	CHEMBL308279	0,842	0,732	1,519	0,913	0,606	GI50 = 52.72 nM	GI50 = 1000.0 nM	1
**DB12359**	CHEMBL3891745	0,98	0,759	1,518	0,772	0,746	GI50 = 610.0 nM	GI50 = 870.96 nM	1
**DB12570**	CHEMBL3586565	0,859	0,743	1,513	0,884	0,629	IC50 = 38.0 nM		1

Table S7. DrugBank ligands with reported activity on PC cell lines identified from the similarity screening, which did not result as particularly promising for repurposing on PC according to the performed analyses and literature data.

Molecule Name	2D structure	Activity *in vitro*	Activity *in vivo*	Max phase	Clinical trial /Patent	Disease	Primary target
**Zebularine**	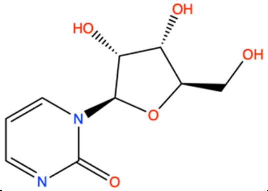	Doubling time of PC-3 cells is almost doubled 39hrs to 59hrs		0			
**Gimatecan**	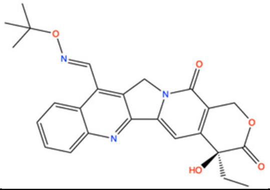	IC50 = 0.0026µg/ml (PC-3); IC50 = 0.0034 µg/ml (DU-145)		2		Breast cancer (Phase 2)	DNA topoisomerase I (TOP1) [Inhibitor]
**Lurtotecan**	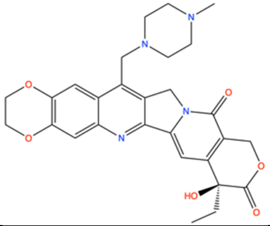	IC50 = 1.61 ng/ml (DU-145); IC50 = 0.44 ng/ml for liposomal formulation (DU-145)		2		Ovarian cancer (Discontinued in Phase 2); Lung cancer (Discontinued in Phase 1)	DNA topoisomerase I (TOP1) [Inhibitor]
**Belotecan**	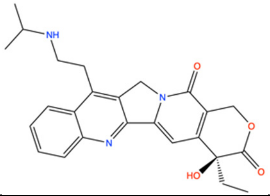				Phase I on 2 PC patients	Solid tumour/cancer (Phase 2)	DNA topoisomerase I (TOP1) [Inhibitor]
**Demecolcine**	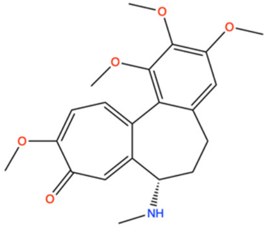	Activity = 31 nM (PC-3)		0		Solid tumour/cancer (Approved)	Microtubule-associated protein (MAP) [Modulator]

**Verubulin**	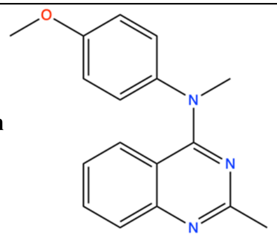		2	Phase I on 3 PC patients	Solid tumour/cancer (Phase 2); Brain metastases (Phase 2); Glioblastoma multiforme (Phase 2); Recurrent glioblastoma (Phase 2)	
**K-00546**	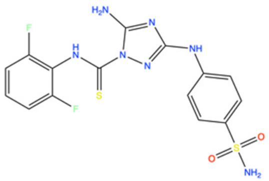	Tested on PC-3, (data not shown)	0			Cyclin-dependent kinase 1 (CDK1) [Inhibitor]; Cyclin-dependent kinase 2 (CDK2) [Inhibitor]; VEGFR1 messenger RNA (VEGFR1 mRNA) [Inhibitor]
**Oleandrin**	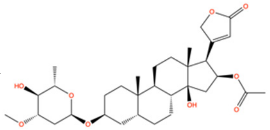	IC50 = 0.001 µg/ml (PC-3); IC50 = 0.002 µg/ml (DU-145)	1		Solid tumour/cancer (Phase 2); Pancreatic cancer (Phase 2)	Sodium/potassium-transporting ATPase (SPT ATPase) [Inhibitor]
**Tezacitabine**	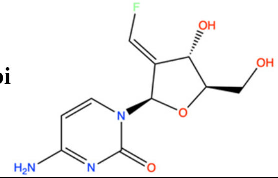	Cell viability decrease at 10µM (PC-3)	2		Gastric adenocarcinoma (Discontinued in Phase 2)	Ribonucleoside-diphosphate reductase M2 (RRM2) [Modulator]
**Dichlororibofuranosylbenzimidazole (DRB)**	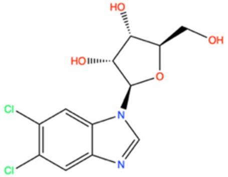	Apoptotic effect on PC-3 cells cotreated with API-2	0			Casein kinase II alpha (CSNK2A1) [Inhibitor]
**Annamycin**	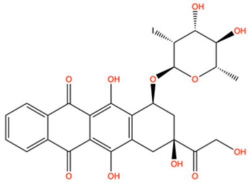	Tested on LNCaP-Pro5 cells, (data not shown)	1		Acute myeloid leukaemia (Phase 1/2)	[DNA topoisomerase II alpha (TOP2A); NaN]

**K-252a**	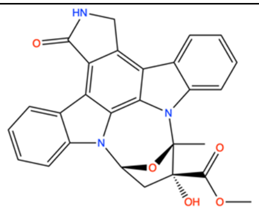	Cell viability = 91% (PC-3), =94% (DU-145) at 100 nM		0		Solid tumour/cancer (Investigative)	[Protein kinase D (PRKD1); Inhibitor]
**4'-Thio-Fac (FF-10502)**	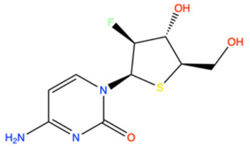			1	Phase I 2 PC patients (NCT02661542)	Solid tumour/cancer (Phase 1/2)	[DNA polymerase beta (POLB); Inhibitor]
**5'-Deoxy-5'-methylthioadenosine**	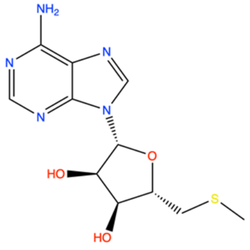		Tumor volume 64% at 100mg/kg (MTA) + 75 mg/kg 6-TG (LTL352)	0		Multiple sclerosis (Terminated)	Adenosine A2b receptor (ADORA2B) [Inhibitor]; Adenosine A2a receptor (ADORA2A) [Inhibitor]; Adenosine A1 receptor (ADORA1) [Inhibitor]; Adenosine A3 receptor (ADORA3) [Inhibitor]; S-methyl-5-thioadenosine phosphorylase (MTAP) [Inhibitor]
**ZD-6126**	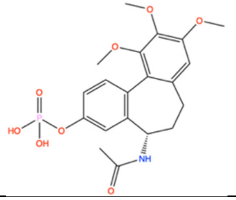		Necrosis >80-90% at 200 mg/kg (PC-3)	2	Halted in phase II for toxic effects on humans	Solid tumour/cancer (Phase 2)	
**Diethylhomospermine (DEHSPM)**	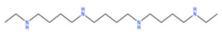	ID50 = 0.7 µM(PC-3); ID50 = 0.03 µM(DU-145); ID50 = 0.1 µM(LNCaP); ID50 = 0.2µM(DuPro)		1	Phase I on 2 PC patients. Halted for toxic effects		
**Bicuculline**	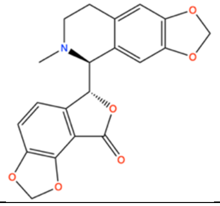	Data not shown, inactive on PC-3, DU-145, LNCaP, MDA-PCA-2B		0			Gamma-aminobutyric acid receptor (GAR); [Antagonist]; Glutamate receptor AMPA (GRIA) [Antagonist]

**Aminopterin**	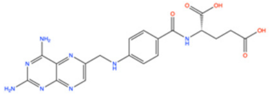		2	PATENT WO9818493A2	Leukaemia (Withdrawn from market)	Polypeptide deformylase (PDF) [Inhibitor]
**Batabulin**	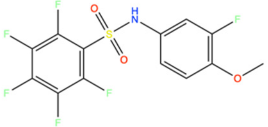		2	PATENT WO2011057064A1	Solid tumour/cancer (Phase 2/3)	
**2-Fluoroadenosine**	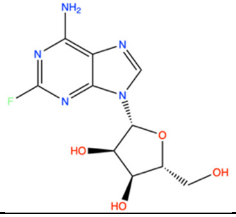		0	PATENT EP2711007		
**AUY922**	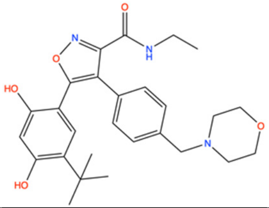		0	PATENT EP2370076A2		
**Lamotrigine**	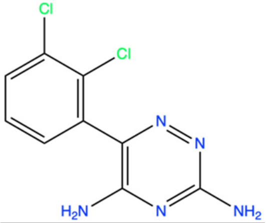		4	Associated with PC risk reduction	Bipolar disorder (Approved); Epilepsy (Approved)	Voltage-gated sodium channel alpha Nav1.9 (SCN11A) [Blocker]
**LY3201**	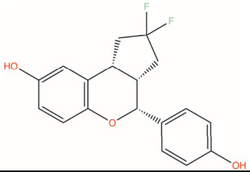	Cellular proliferation decrease from 1µM (LNCaP)	0			Estrogen receptor beta (ESR2) [Agonist]; Estrogen receptor alpha (ESR1) [Agonist]
**S-Equol**	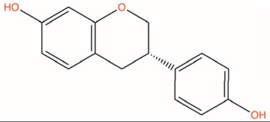	Cell viability decrease at 10µM (LNCaP)	2	NCT00962390	Hot flushes (Phase 2); Alzheimer (Phase 1/2)	Estrogen receptor beta (ESR2) [Agonist]

**Peruvoside**	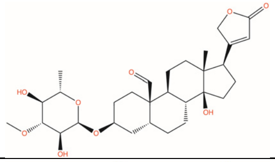	Block of cell growth at 50nM (LNCaP-abl)		0			Cardiac glycoside
**Acadesine**	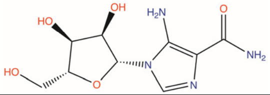	IC50 = 40-50 µM (PC-3, DU-145); IC50 ~ 50 µM (LNCaP)		3		Diabetic complication (Phase 3)	AMP-activated protein kinase (AMPK) [Modulator]
**Cordycepin**	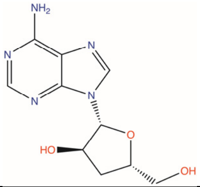	Cell viability decrease from 5µg/ml (PC-3; LNCaP), from 15µg/ml (DU-145)		1			
**Cardamonin**	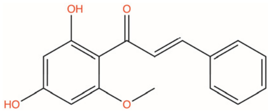	GI50 = 11.35 µg/ml (PC-3); Cell proliferation = 60 % (DU-145), = 80 % (LNCaP) at 10 µM		0			
**Phloretin**	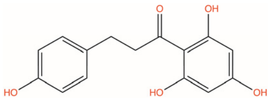	IC50 = 25µM (LNCaP); IC50 = 39.4 µM (PC-3)		0			Aquaporin-9 (AQP9) [Inhibitor]; Chloride channel protein 3 (CLC-3) [Blocker]; Solute carrier family 23 member 1 (SLC23A1) [Inhibitor]
**Crolibulin**	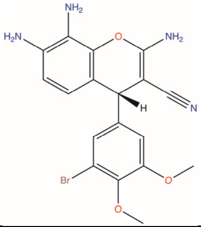			1	Phase I/II in combination with cisplatin NCT01240590	Solid tumour/cancer (Phase 1/2)	
**Fenbendazole**	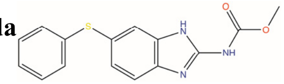	ED50 = 1.82 µM (PC-3); ED50 = 10.4 µM (DU-145)	TCI = 91% with FBZ 100mg/kg (Dunning rat AT6.1)	0			

**Pralatrexate**	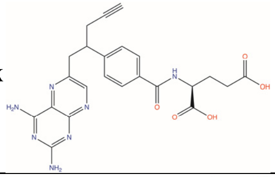	IC50 = 0.01 μM(PC-3); IC50 = 0.015 μM (DU-145)	4		Breast cancer (Approved); Peripheral T-cell lymphoma (Approved)	Polypeptide deformylase (PDF) [Inhibitor]
**Amrubicin**	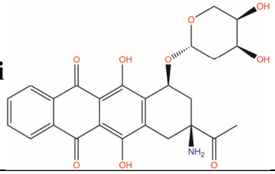	Tested on PC-3 and LNCaP (data not shown)	3	Case report on small cell PC-3	Small-cell lung cancer (Phase 3)	

Table S8. *In vitro* activity records on other tumor cell lines for the 10 candidates proposed for PC repurposing, retrieved from ChEMBL. Only activity data expressed by means of: (i) "Standard Type" equal to GI_{50}, EC_{50}, or IC_{50}; (ii) "Standard Relation" equal to "=", and; (iii) "Standard Unit" in the nanomolar range (i.e., "nM") was retained.

Drug	Disease	Cell line	Activity
**Bimiralisib**	Amelanotic melanoma	A2058	IC50 = 2333 nM
	Ovarian serous cystadenocarcinoma	SKOV3	IC50 = 237 nM
**Onatasertib (CC-223)**	Breast carcinoma	CAL-51	IC50 = 140 nM
	Lung adenocarcinoma	A549	IC50 = 208 nM
	Invasive breast carcinoma of no special type	T47D	IC50 = 92 nM
	Lung large cell carcinoma	NCI-H460	IC50 = 200 nM
	Hepatoblastoma	HepG2	IC50 = 321 nM
	Breast adenocarcinoma	AU565	IC50 = 329 nM
	Childhood hepatocellular carcinoma	Hep3B	IC50 = 338 nM
	Glioblastoma	U87MG	IC50 = 555 nM
	Colon carcinoma	HCT116	IC50 = 371 nM
	Breast adenocarcinoma	MDA-MB-231	IC50 = 669 nM
	Lung adenocarcinoma	NCI-H23	IC50 = 1039 nM
**VS-5584**	Childhood acute monocytic leukemia	MV4-11	IC50 = 200 nM
	Invasive breast carcinoma of no special type	MCF7	IC50 = 130 nM
**BIIB021**	Invasive breast carcinoma of no special type	MCF7	IC50 = 100 nM
	Colon carcinoma	HCT116	GI50 = 150 nM
	Lung adenocarcinoma	NCI-H1975	GI50 = 200 nM
	Lung large cell carcinoma	NCI-H460	GI50 = 210 nM
	Childhood hepatocellular carcinoma	Hep3B	GI50 = 240 nM
	Breast adenocarcinoma	MDA-MB-231	GI50 = 240 nM
	Lung adenocarcinoma	A549	GI50 = 260 nM
	Breast adenocarcinoma	SK-BR-3	GI50 = 347 nM
	Adult acute myeloid leukemia	HL60	GI50 = 590 nM
**Adapalene (CD-271)**	Colon adenocarcinoma	SW480	IC50 = 405 nM
	Colon carcinoma	HCT116	IC50 = 597 nM
	Colon adenocarcinoma	SNU-C1	IC50 = 1779 nM
**Picropodophyllin**	Lung adenocarcinoma	A549	IC50 = 60 nM
**VS-4718**	Pancreatic ductal adenocarcinoma	BXPC-3	IC50 = 930 nM
	Lung adenocarcinoma	NCI-H1975	IC50 = 340 nM
	Breast adenocarcinoma	MDA-MB-231	IC50 = 29 nM

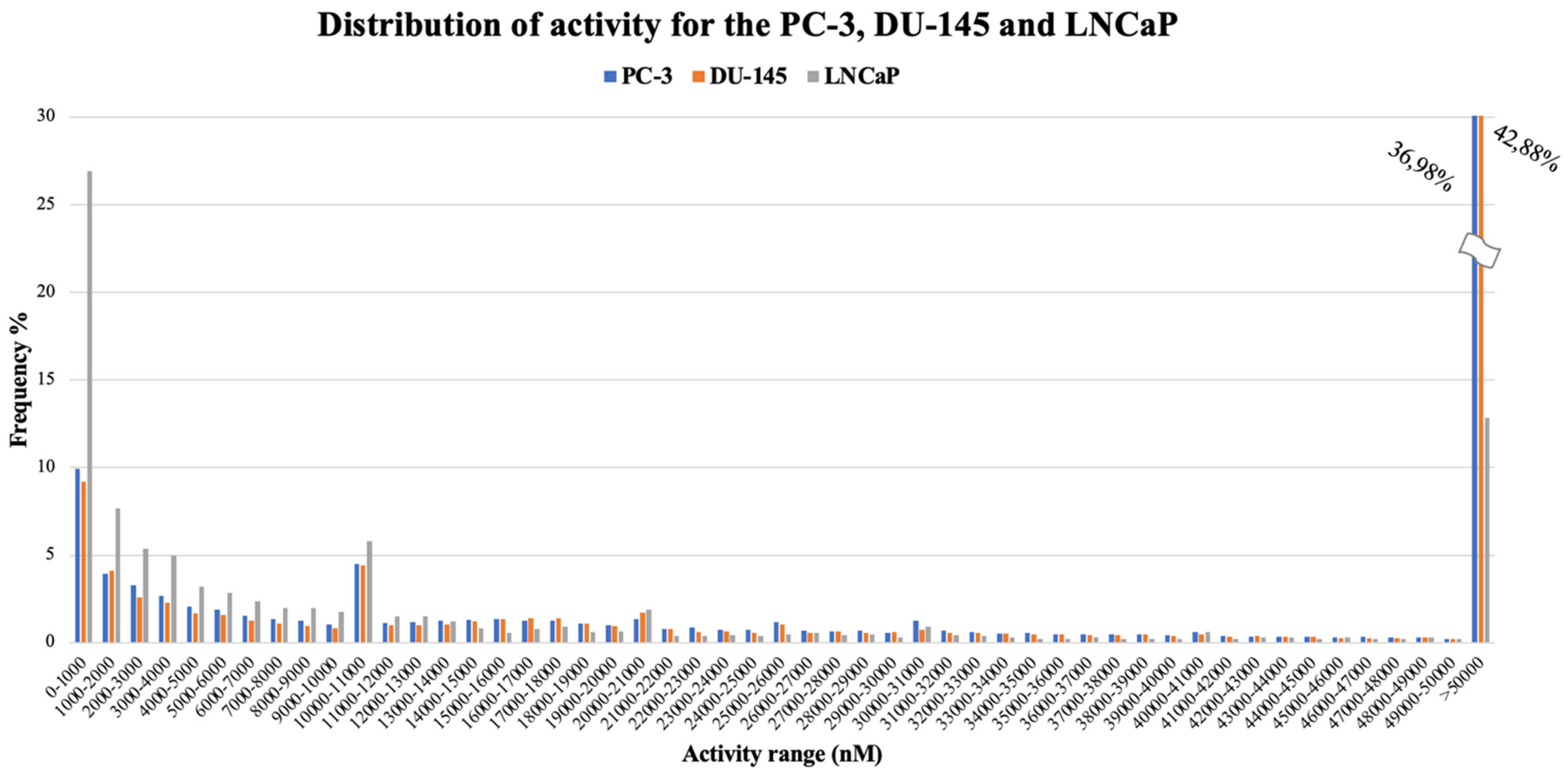

Figure S1. Activity values distribution for the molecules tested on PC-3, DU-145 and LNCaP PC cell lines reported on ChEMBL.

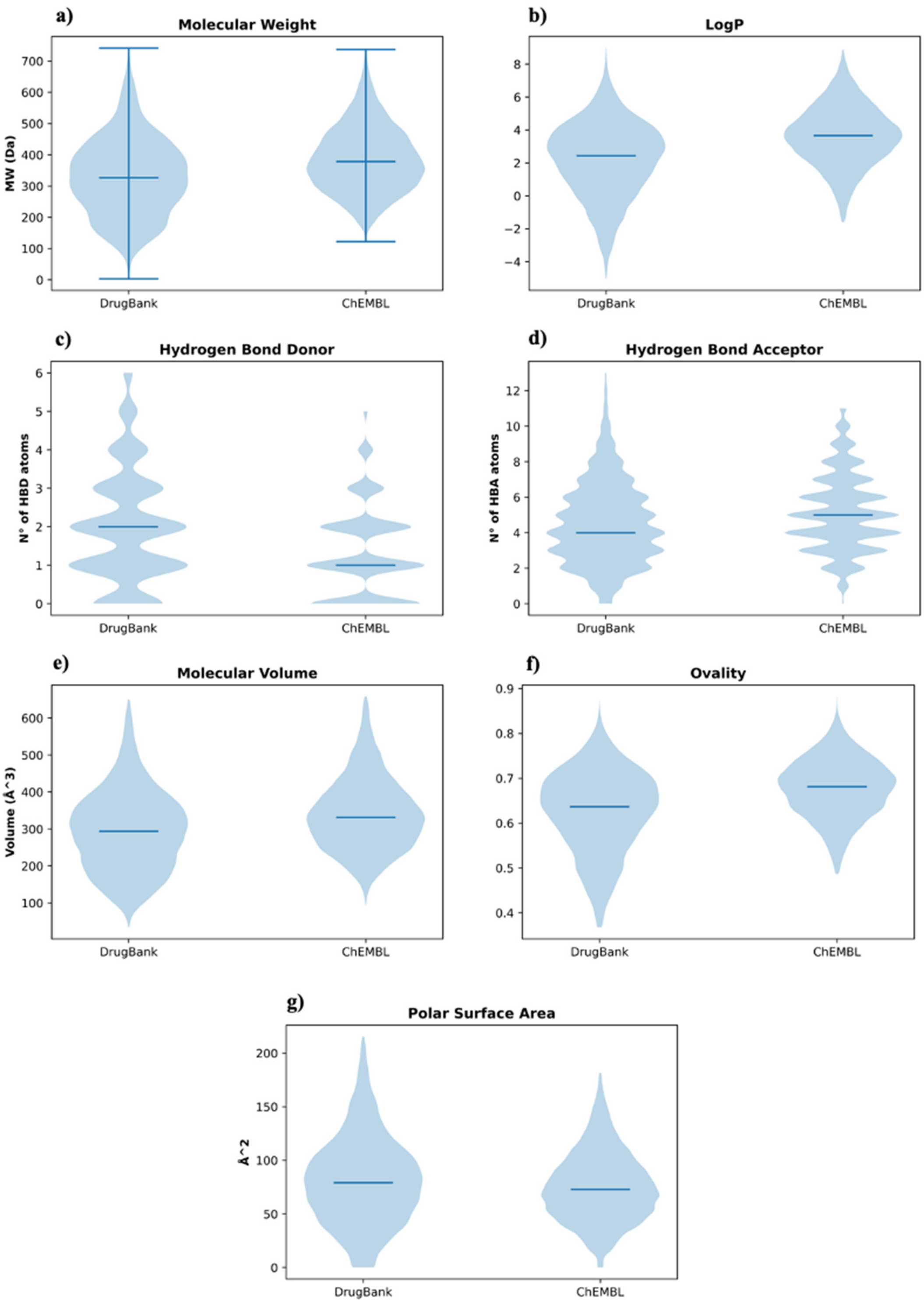

Figure S2. Distribution of molecular descriptors for the molecules of the DrugBank and ChEMBL datasets: **a)** molecular weight; **b)** LogP; **c)** Hydrogen bond donor atoms; **d)** Hydrogen bond acceptor atoms; **e)** molecular volume (VABC Volume); **f)** ovality; **g)** polar surface area. All molecular descriptors were calculated with KNIME nodes "RDKit Descriptor Calculation" and "CDK Molecular properties". Ovality was obtained with the formula $O = \frac{A}{4\pi(\frac{3V}{4\pi})^{2/3}}$. Outliers below 1st quartile and above 3rd quartile were removed prior plots generation to highlight differences between the two datasets.

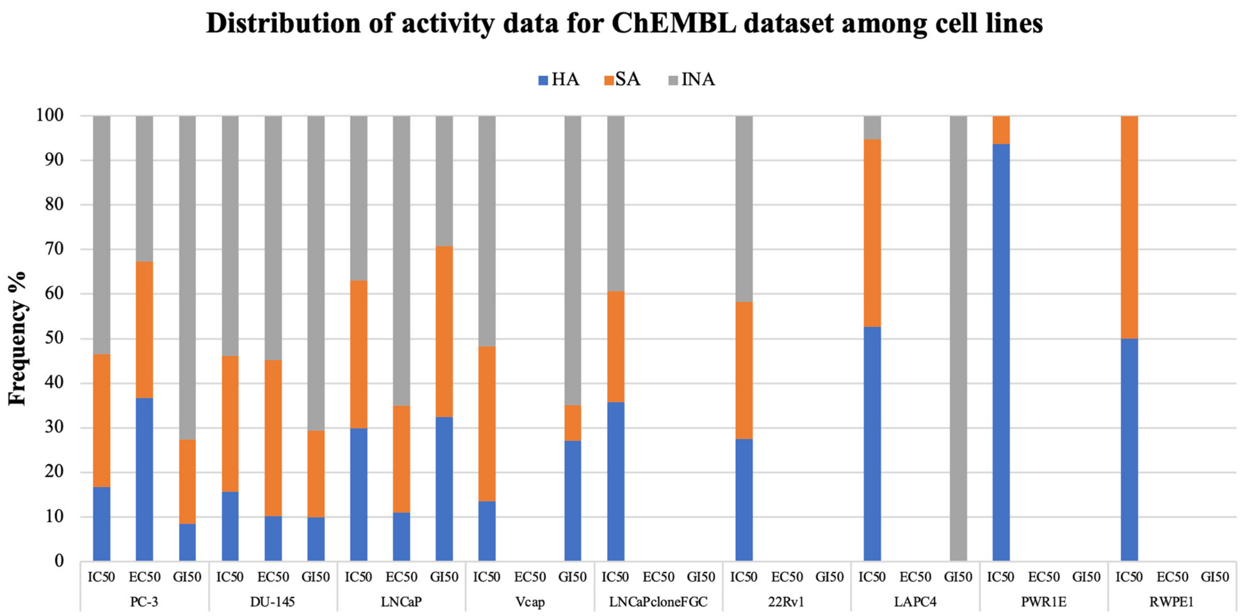

Figure S3. Bar plot of the activity data for the PC cell in the "*Standard Types*" IC_{50}, EC_{50}, or GI_{50}. In this study, we considered: i) "*highly active*" molecules (HA), those with an activity value on PC cell lines below 1 µM; ii) "*scarcely active*" molecules (LA) with activity values on PC cells higher than 1 µM, and lower or equal to10 µM, and; iii) inactive molecules (INA) with activity values against PC cells higher than 10 µM. Only the cell lines PC-3, DU-145, and LNCaP have activity records for all three "*Standard Types*" with comparable distribution of the records inside the considered ranges.

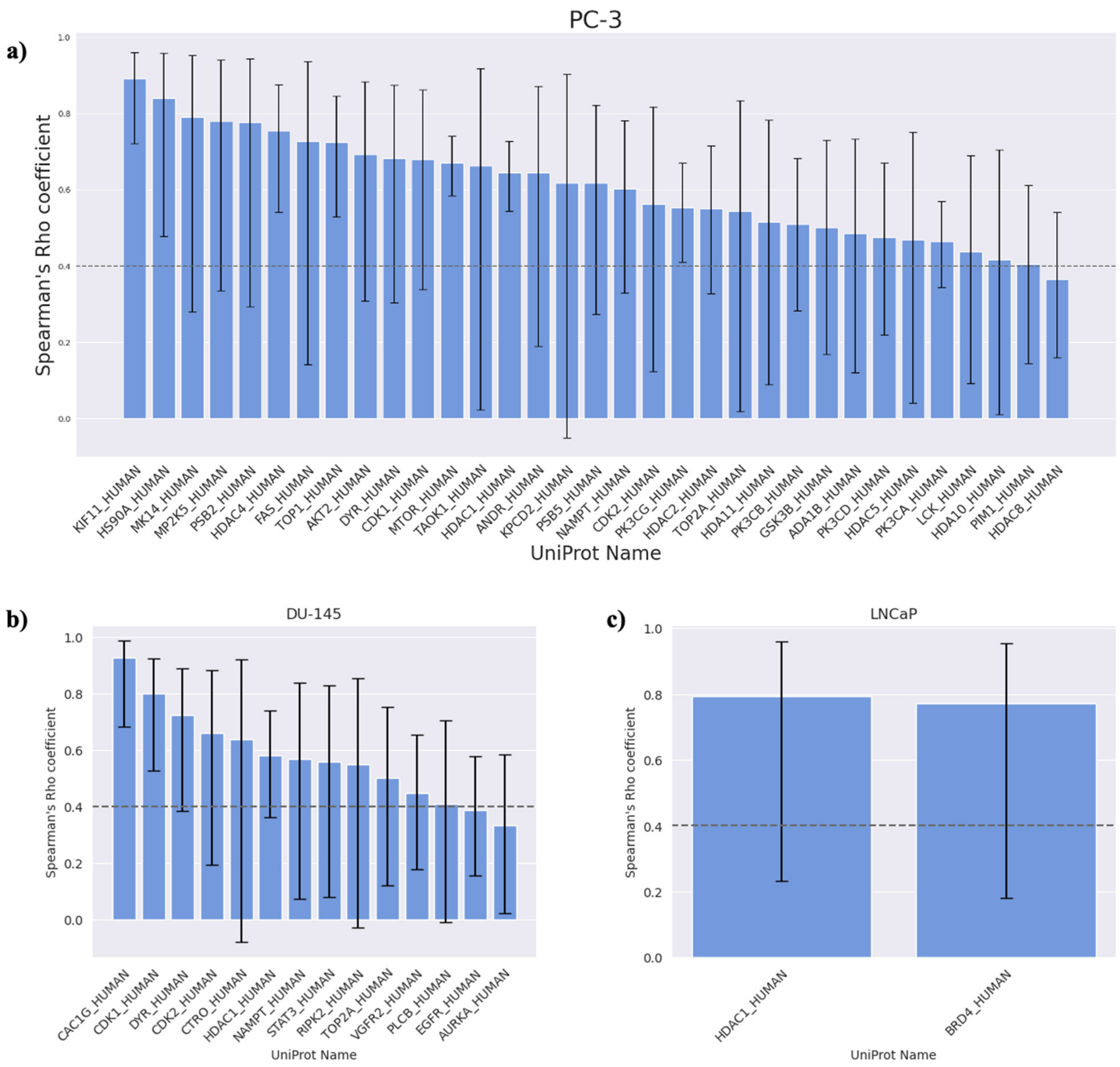

Figure S4. Evaluated Spearman's Rho correlation coefficient of correlation (ρ_s) with their intervals of confidence. All *p*-values are < 0.05 (see **Table S4**), $\rho_s \geq 0.4$ was the threshold considered for an acceptable degree of correlation. The three panels represent the targets divided on the cell line of identification: **a)** PC-3, **b)** DU-145 and **c)** LNCaP cells.

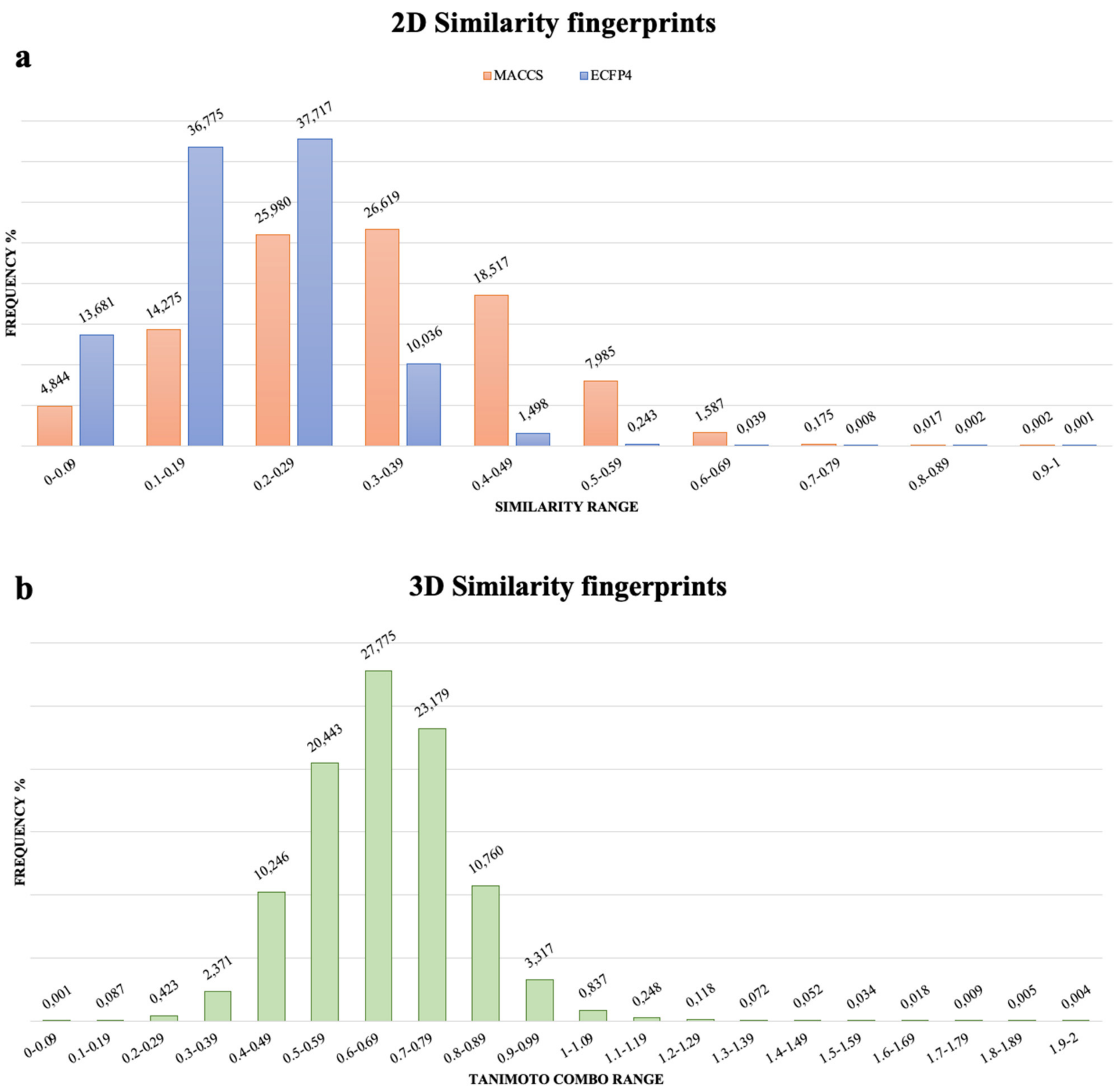

Figure S5. Frequency distributions of the estimated ligand-based similarities. In particular, panel **a)** reports the frequency distribution of the MACCS and ECFP4 fingerprints values obtained from the 2D similarity estimations on ChEMBL compounds versus DrugBank molecules. Panel **b)** reports the frequency distribution of the Tanimoto Combo scores, obtained at the 3D shape estimations performed on similar ChEMBL *versus* DrugBank molecules.
